# Evolution patterns and characteristics of ballasted track bed lateral resistances in cold regions under progressive freezing of ice layers

**DOI:** 10.1371/journal.pone.0356815

**Published:** 2026-08-27

**Authors:** Jianxing Liu, Wei Qi, Aiping Chen, Yong Cao, Lei Kou, Mykola Sysyn, Zhiwei Ma

**Affiliations:** 1 Sichuan Provincial Engineering Research Center of Rail Transit Lines Smart Operation and Maintenance, Chengdu Vocational and Technical College of Industry, Chengdu, Sichuan, China; 2 School of Civil Engineering, Southwest Jiaotong University, Chengdu, Sichuan, China; 3 School of Qilu Transportation, Shandong University, Jinan, Shandong, China; 4 Department of Planning and Design of Railway Infrastructure, Technical University Dresden, Dresden, Saxony, Germany; China Construction Fourth Engineering Division Corp. Ltd, CHINA

## Abstract

The freezing process of ballasted track beds in cold regions is a dynamically evolving phenomenon. Co-regulated by factors such as ice content, freezing temperature, and freezing zone, this process exacerbates the complexity of the evolution of ballast bed lateral resistance. To address this, a combination of full-scale ballast bed lateral resistance tests, uniaxial compression tests, the discrete element method (DEM), and response surface optimization design (D-optimal design) was employed to investigate the macro-mesoscopic impacts of progressive ice layer freezing on the evolutionary process of ballast bed lateral resistance. Firstly, a full-scale ballast bed was constructed to conduct lateral resistance tests. Subsequently, ballast-ice composites were prepared under various temperatures to perform uniaxial compression tests. Following this, a DEM model of the ballasted bed was established, into which a parallel-bond model was incorporated to simulate the ballast-ice composites. Both types of DEM models were validated through comparison with experimental results. A set of progressive freezing conditions was then designed using the D-optimal method. Combined with the DEM simulation results of the ballast bed resistance under different freezing conditions, a predictive model for the lateral resistance of the ballasted bed was established, coupling ice content, freezing temperature, and freezing depth. Concurrently, based on the DEM models under varying freezing conditions, the mesoscopic mechanism of how those three factors affect the evolutionary characteristics of the lateral resistance was analyzed. The results indicate that ice content and freezing depth are the dominant factors influencing the lateral resistance of the ballasted bed, both exhibiting a significant positive correlation. The influence of temperature is relatively weak but manifests indirectly through interactive effects. Mesoscopic analysis reveals that progressive ice layer freezing inhibits particle movement within the ballast bed by increasing the number of ballast-ice parallel bonds, elevating the particle coordination number, and strengthening the force chain network. Consequently, the ballast bed transforms from a friction-dominated loose system into a cohesive-frictional composite stable system. The findings of this study elucidate the evolutionary patterns and mesoscopic strengthening mechanisms of ballast bed lateral resistance under progressive ice layer freezing. Hoping those findings can provide a theoretical foundation for the design, maintenance, and service performance evaluation of ballasted tracks in cold regions.

## 1. Introduction

As one of the most prominent structural forms in railway systems, the ballasted track exhibits distinct advantages, including excellent vibration reduction, superior drainage capacity, low initial construction costs, and high structural adjustability [1,2]. These merits have led to its widespread application worldwide, while also offering notable operational advantages in railway projects characterized by complex topography and harsh climatic environments. As the most characteristic component of ballasted tracks, the ballast bed bears and transmits various vertical, lateral, and longitudinal forces transferred from the sleepers. This is accomplished through the internal friction and interlocking behaviors among individual ballast particles, thereby providing stable support and confinement for the track superstructure [3,4]. To investigate the mechanical properties and evolutionary patterns of the ballast layer, researchers commonly rely on individual or collective particle-level characterizations, such as crushing tests [5], Los Angeles abrasion tests [6], static or dynamic direct shear tests [7,8], and triaxial tests [9]. Furthermore, macroscopic methodologies evaluating the overall performance of ballast bed were extensively implemented, such as the single sleeper push tests (SSPT) [10,11] and panel pull tests [12]. Most of these experimental studies were conducted under normal temperature conditions, which is determined by the operating environment of most ballasted tracks. Nonetheless, certain railway lines traverse cold regions where the ballasted track must endure severe challenges from snow accumulation and sub-zero temperatures. These lines are predominantly distributed in Norway, Sweden, Switzerland, Finland, Russia, and Japan, as well as the Qinghai-Tibet Railway in China and the Alaska Railroad in the United States [2,13,14].

Due to their unique geographical environments, railways in cold regions present severe challenges to the ballast beds under extreme climatic conditions. Particularly during late winter and early spring, frost and snow initially cover the surface of the ballast bed, leading to the freezing of the aggregate in the upper ballast layer. As ambient temperatures rise, the surface ice and snow partially melt, subsequently infiltrating the ballast bed through the interconnected voids among individual ballast particles. When ambient temperatures drop again, this meltwater refreezes into ice, which not only bonds the ballast particles into consolidated blocks but also adheres to the particle surfaces, thereby reducing the inter-particle friction coefficient. Furthermore, during rainy seasons, the cold and wet climate can induce freezing rain—a meteorological phenomenon where supercooled raindrops rapidly freeze upon contact with the ground or exposed objects [15]. Because freezing rain consists of supercooled liquid droplets rather than solid ice pellets, it can penetrate into the surface layer and inner zones of the ballast bed to varying extents, forming ice layers within the ballast skeleton. Under the recurrent, synergistic effects of frost, rain, snow, and temperature fluctuations, the ballast bed undergoes freezing phenomena characterized by distinct ballast-ice bonding strengths and depths. These ice layers restrict the inherent contact force transmission mechanisms and spatial mobility among ballast particles to varying degrees, ultimately leading to significant alterations in the mechanical properties of the ballast bed.

The progressive freezing of ice layers poses a significant threat to the stability and durability of the ballast bed. In fact, with the long-term operation and continuous construction of ballasted tracks in alpine and severe cold regions, the service performance of the ballast bed under low-temperature freezing environments has progressively garnered the attention of researchers and engineers. Nurmikolu [16,17] and Guthrie [17] elucidated the adverse impacts of Finland’s cold climate—particularly in areas prone to seasonal frost action—on the ballast beds of high-speed railways, accentuating that understanding and accounting for frost action mechanisms plays a pivotal role in the successful construction and maintenance of high-speed rail lines. Through field observations of several ballasted tracks in China, Sheng et al. [18] documented that the freezing of the ballast layer is a pervasive phenomenon even within clean ballast beds with low moisture content. Similarly, by monitoring railway tracks in northern Japan, Akagawa et al. [19] discovered that track heaving in severely cold regions originates not only from the frost heave of the subgrade layer but also from that of the ballast layer itself. Guo et al. [20] recognized the freezing and frost-heaving phenomena in the ballast layer, utilizing laboratory image analysis to track and qualitatively characterize the ice growth and ballast particle movement during the cooling process. Ižvolt et al. [21,22] constructed a 1:1 full-scale outdoor ballasted track test rig to monitor and comparatively analyze the freezing behavior of ballasted tracks during winters in Slovakia and Lithuania. Subsequently, they conducted numerical simulations of frozen ballast beds using SoilVision software and proposed a modified nomogram design to determine the required thickness of subgrade surface layers susceptible to frost action. Liu et al. [23] integrated uniaxial compression tests of frozen ballast with the discrete element method (DEM) to explore the compressive performance of ballast aggregates in freezing environments, revealing that their compressive capacity is closely linked to variations in temperature and freezing strength. Furthermore, Li et al. [24], employing the same experimental methodology, further analyzed the influences of ice content and temperature on the compressive properties of frozen ballast layers. Their results indicated that an increase in ice content enhances the ductile characteristics of the frozen specimens, causing their failure mode to transition from brittle failure to axial splitting failure. To qualitatively evaluate the impact of freezing environments on ballast bed resistance, Liu et al. [25] constructed a full-scale ballasted track model inside a large-scale environmental chamber and conducted longitudinal and lateral resistance tests on the frozen ballast bed under various low-temperature conditions. The findings demonstrated that the freezing environment increases the resistance of the ballast bed at small sleeper displacements; however, once the displacement exceeds a certain threshold, the ballast bed resistance decreases. Li et al. [26] employed DEM combined with a bonding model to further investigate the mesoscopic resistance performance of frozen ballast beds, systematically analyzing the influences of variations in ballast shoulder height, shoulder width, and crib/shoulder slope on the resistance of frozen ballast beds.

The above studies have explored the performance of the ballast bed in freezing environments through field monitoring, laboratory testing, and numerical simulations, which yielding invaluable results and experience. However, the infiltration and freezing of ice within the ballast layer is a progressive process, during which the degree of freezing between ballast particles is significantly co-regulated by the ice content, freezing temperature, and frozen zones within the ballast profile. Notably, while existing literatures (e.g., [23,24]) have focused on the compressive performance of frozen ballast layers under varying ice contents and temperatures, the specific influence of ice distribution dynamics on the resistance performance of the ballast layer remains elusive. Furthermore, the synergistic effects of ice content, freezing temperature, and frozen zones during the progressive freezing of ice layers inevitably exacerbate the complexity of the mechanical properties of the ballast layer. Thus, this study integrates full-scale ballast bed lateral resistance tests, uniaxial compression tests on ballast-ice composites, frozen ballast bed DEM simulations, and a D-optimal response surface design to systematically investigate the dynamic evolution of ballast bed lateral resistance under progressive ice freezing conditions. Then, a predictive model for lateral resistance coupled with three factors—ice content, freezing temperature, and freezing depth—was established, achieving an effective linkage between macro- and mesoscopic multiscale analyses. Meanwhile, from a mesoscopic perspective, the strengthening mechanism characterized by an increased number of ice-ballast parallel bonding keys, elevated particle coordination numbers, and reinforced force chain networks was revealed, clarifying the intrinsic mechanism driving the transition of the ballast bed from a friction-dominated loose system to a cohesive-frictional composite stable system.

## 2. Materials and laboratory experiments

### 2.1. Ballast aggregates

**Fig 1:**
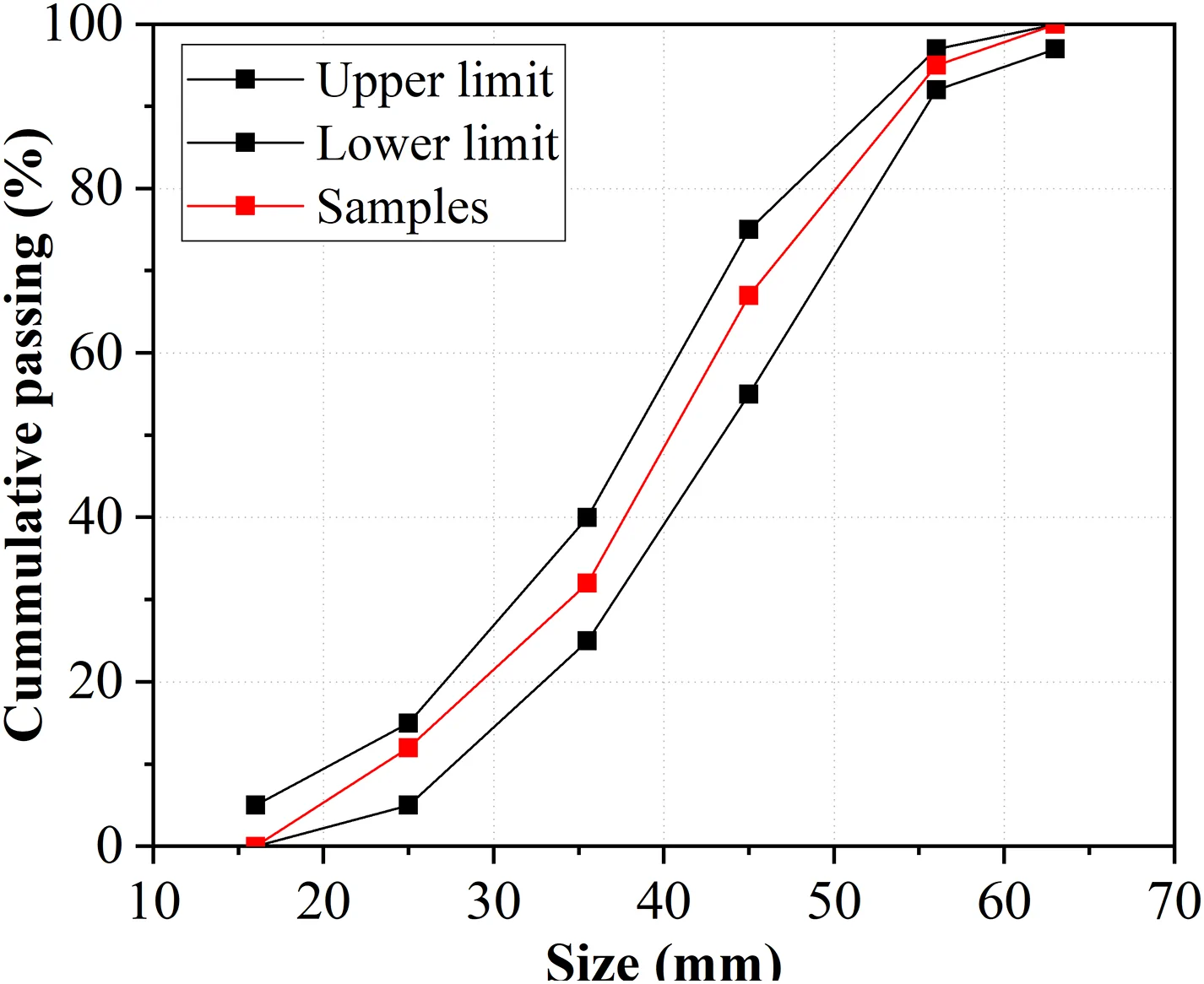
Particle size distribution.

China’s newly constructed Class I railway ballast was selected as the research object. According to the particle size distribution (PSD) curve of Class I ballast specified in Railway Ballast (TB/T 2140−2008) [27], ballast particles within various size fractions were prepared via mechanical sieving. The PSD curve of the ballast aggregates is shown in Fig 1.

### 2.2. Sleeper and ballast bed

**Fig 2:**
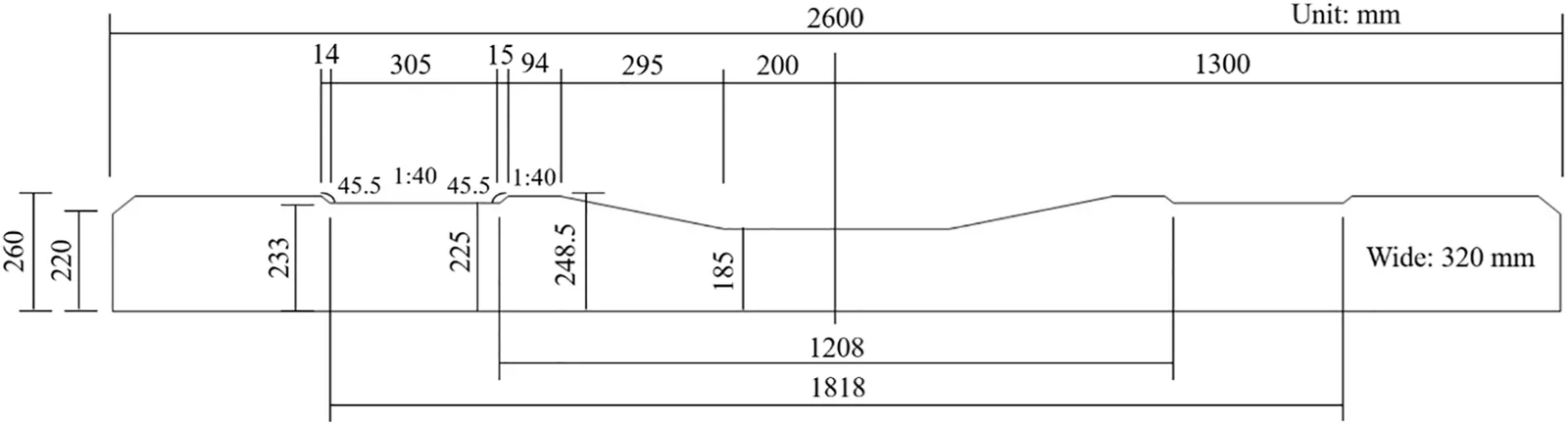
Size of China’s Type III prestressed concrete sleeper.

**Fig 3:**
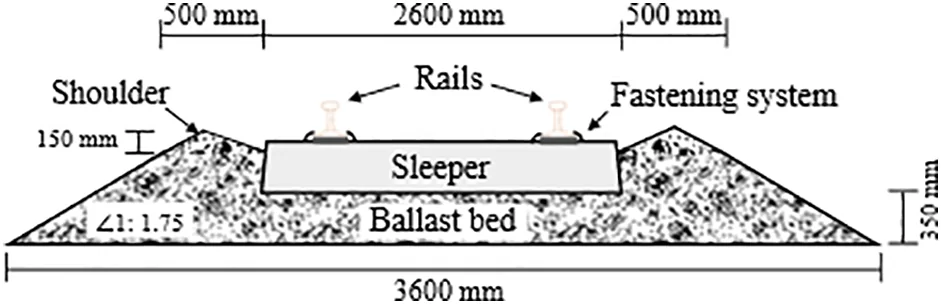
Size of ballast bed.

Firstly, a China’s Type III prestressed concrete sleeper (Fig 2) was selected, with a length of 2600 mm, a width of 320 mm, an end height of 260 mm, a mid-span height of 185 mm, and a total mass of 340 kg. Subsequently, the ballast bed was constructed using the sieved Class I ballast aggregates. The experimental ballast bed model (Fig 3) was 5 m in length, 3.6 m in width, and 0.35 m in thickness, featuring a shoulder slope of 1:1.75 and a sleeper spacing of 0.6 m. To ensure structural stability, multiple rounds of tamping and compaction were executed both before and after the placement of the sleeper, followed by laying the ballast surface layer. Specifically, the ballast bed was constructed using the layered filling method (with each layer 100−150 mm thick), and each layer underwent 3 rounds of tamping and stabilization operations. The equipment used was the ND-4 handheld internal combustion tamping machine and the DHJ electric compactor. Finally, a stabilized ballast bed was achieved with a bulk density of no less than 1700 kg/m^3^ and two ballast shoulders both height of 0.15 m.

### 2.3. Tests of ballast bed lateral resistances

**Fig 4:**
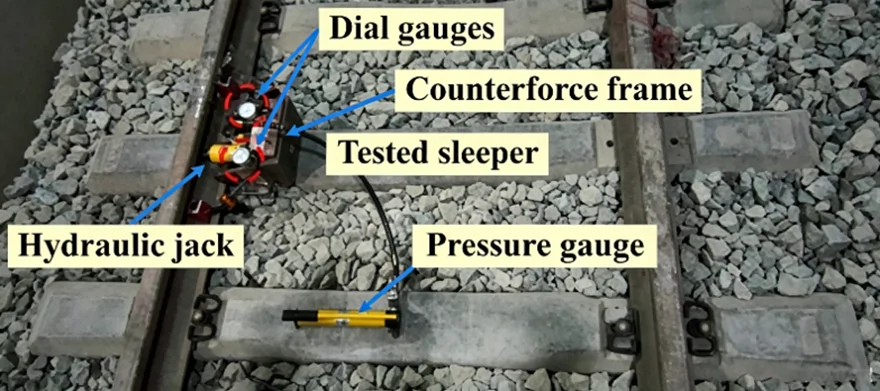
Lateral resistance test of the ballast bed with concrete sleeper.

As is widely acknowledged, the single sleeper push test (SSPT) represents one of the most adopted methodologies for evaluating ballast bed resistance, particularly during in-situ field investigations [28–30]. The underlying mechanism of the SSPT relies on utilizing the track panel frame as a reaction support. Benefiting from its multi-sleeper configuration, the track panel frame can be idealized as a fixed constraint relative to the individual sleeper under investigation. During the loading process, the fastening system on the tested sleeper is dismantled to ensure its complete decoupling from the track superstructure. In this paper, the lateral SSPTs on the ballast bed were executed using a hydraulic jack, dial gauges, and customized test fixtures, as shown in Fig 4.

### 2.4. Preparations of ballast-ice specimens and ice specimens

**Fig 5:**
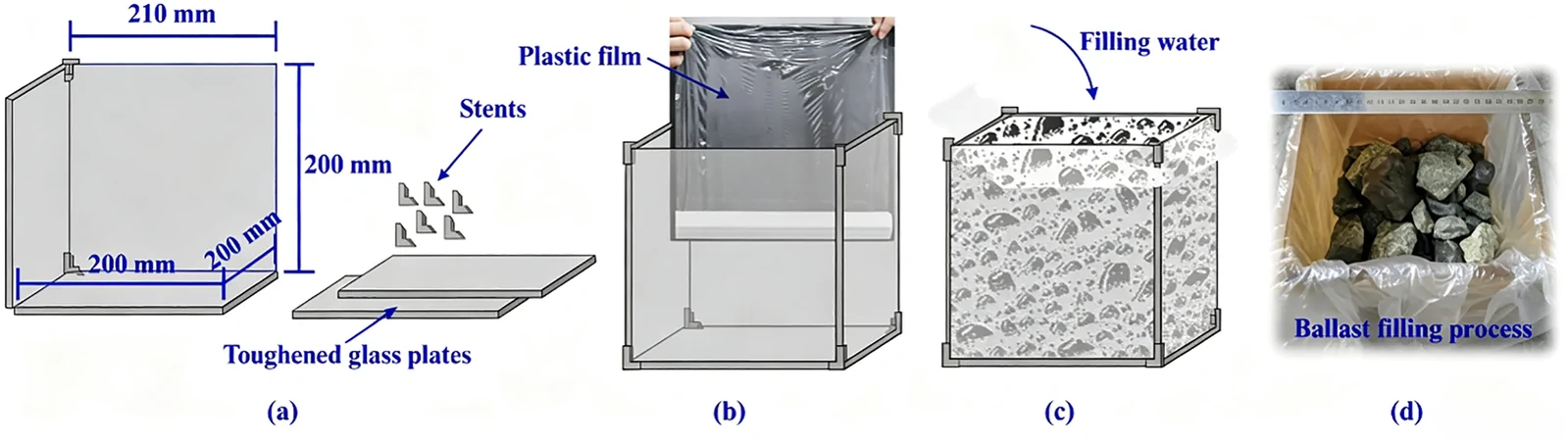
Mold for making ballast-ice composites.

The authors previously developed a preparation methodology for ballast-ice composites [23]. A demountable mold with net internal dimensions of 200 mm was assembled using five tempered glass plates, each featuring dimensions of 210 mm in length, 200 mm in width, and 10 mm in thickness. These glass plates were supported and interlinked via specialized brackets and connectors. To prevent direct contact between the specimen and the mold while ensuring watertightness to eliminate leakage, the inner walls of the mold were lined with an ultra-thin plastic membrane, as illustrated in Fig 5.

To fabricate the ballast-ice composites, the ballast aggregates were first filled into the mold cavity, where they were thoroughly vibrated and compacted. The packing bulk density was determined via the mass-to-volume ratio. If the density failed to meet the minimum threshold of 1.70 g/cm^3^ as stipulated for Class I China’s railway ballast beds [31], the specimen preparation was restarted. Subsequently, the mold was fully inundated with water, and the exact volume of the injected water was recorded. Finally, the assembled specimens were transferred into a temperature-controlled environmental chamber to undergo cooling sequences at targeted temperatures (−10°C, −20°C, and −30°C) and maintained at a constant temperature for 36 hours. Ice specimens with dimensions of 100 mm × 100 mm × 100 mm were prepared by the same method.

### 2.5. Uniaxial compression tests

**Fig 6:**
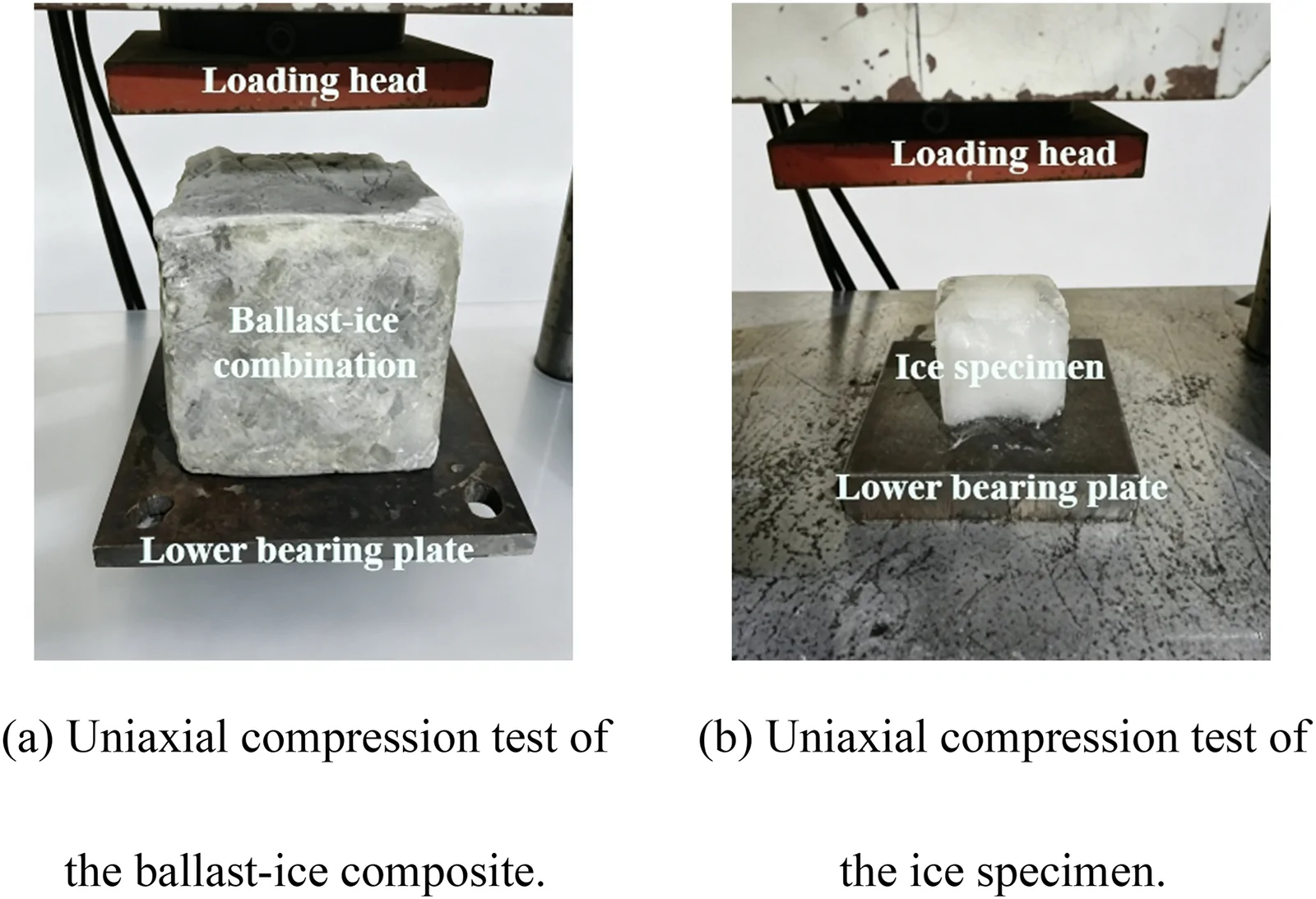
Uniaxial compression test of specimens.

Prior to testing, the mass of each ballast-ice composite was measured to subsequently determine the ice content within the ballast structure. Uniaxial compression tests were then performed on both the pure ice specimens and the ballast-ice composites. The loading scheme was designed with reference to the Standard for Test Method of Mechanical Properties on Ordinary Concrete (GB/T 50081−2002) [32] and Reference [23]. Specifically, a constant stress-controlled loading rate of 0.03 MPa/s was applied to the specimens. This corresponded to a constant force loading rate of 0.03 MPa/s×(0.1 × 0.1) m^2^ = 0.3 kN/s for the pure ice specimens, and 0.03 MPa/s×(0.2 × 0.2) m^2^ = 1.2 kN/s for the ballast-ice composites. Throughout the compression process, liquid nitrogen was used to cool the specimens and their immediate surroundings, thereby maintaining the targeted low-temperature environment. The experimental setup and loading process are shown in Fig 6.

## 3. DEM models

### 3.1. Simulations of lateral resistance of ballast bed

**Fig 7:**
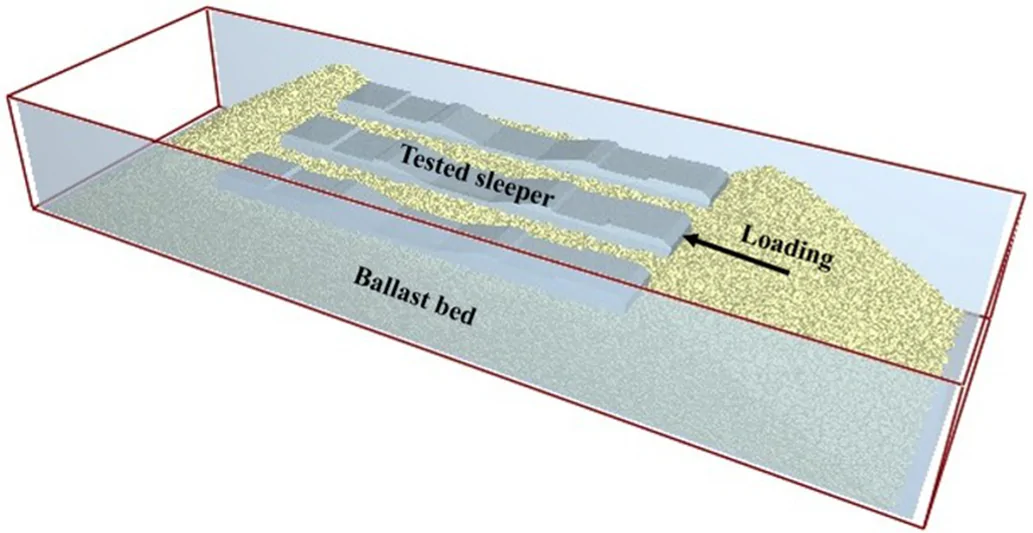
DEM model and lateral resistance tests of the ballast bed.

The three-dimensional (3D) laser scanning technology was utilized to capture the realistic geometry of individual ballast particles, which were subsequently reconstructed via the clump method [33]. According to Reference [33], 8–10-ball clumps were employed to simulate the shapes of ballasts, which balancing computational efficiency and accuracy. In accordance with the PSD curve, the DEM model of the ballast bed was established using a layered compaction method (the specific simulation parameters are listed in Table 1). It should be noted that the sleeper was simulated as a rigid body. Furthermore, two sleepers were respectively placed adjacent to the front and rear of the central tested sleeper, to mitigate the boundary effects on the simulation results. Subsequently, the same loading as applied in the laboratory experiments was imposed onto the tested sleeper within the DEM model, through which the corresponding lateral forces and displacements of the tested sleeper were captured (Fig 7).

**Table 1:** DEM simulation parameters of ballast and sleeper.

Types	Density	Elastic modulus	Poisson’s ratio	Restitution coefficient	Static friction coefficient	Rolling friction coefficient
**Ballast**	2600 kg/m^3^	5.00 × 10^10^ Pa	0.24	0.73	0.58	0.30
**Sleeper**	2000 kg/m^3^	3.60 × 10^10^ Pa	0.25	0.75	0.91	0.67

### 3.2. Establishment of DEM models of ice bodies and ballast-ice composites

#### 3.2.1. Parallel bonding model.

**Fig 8:**
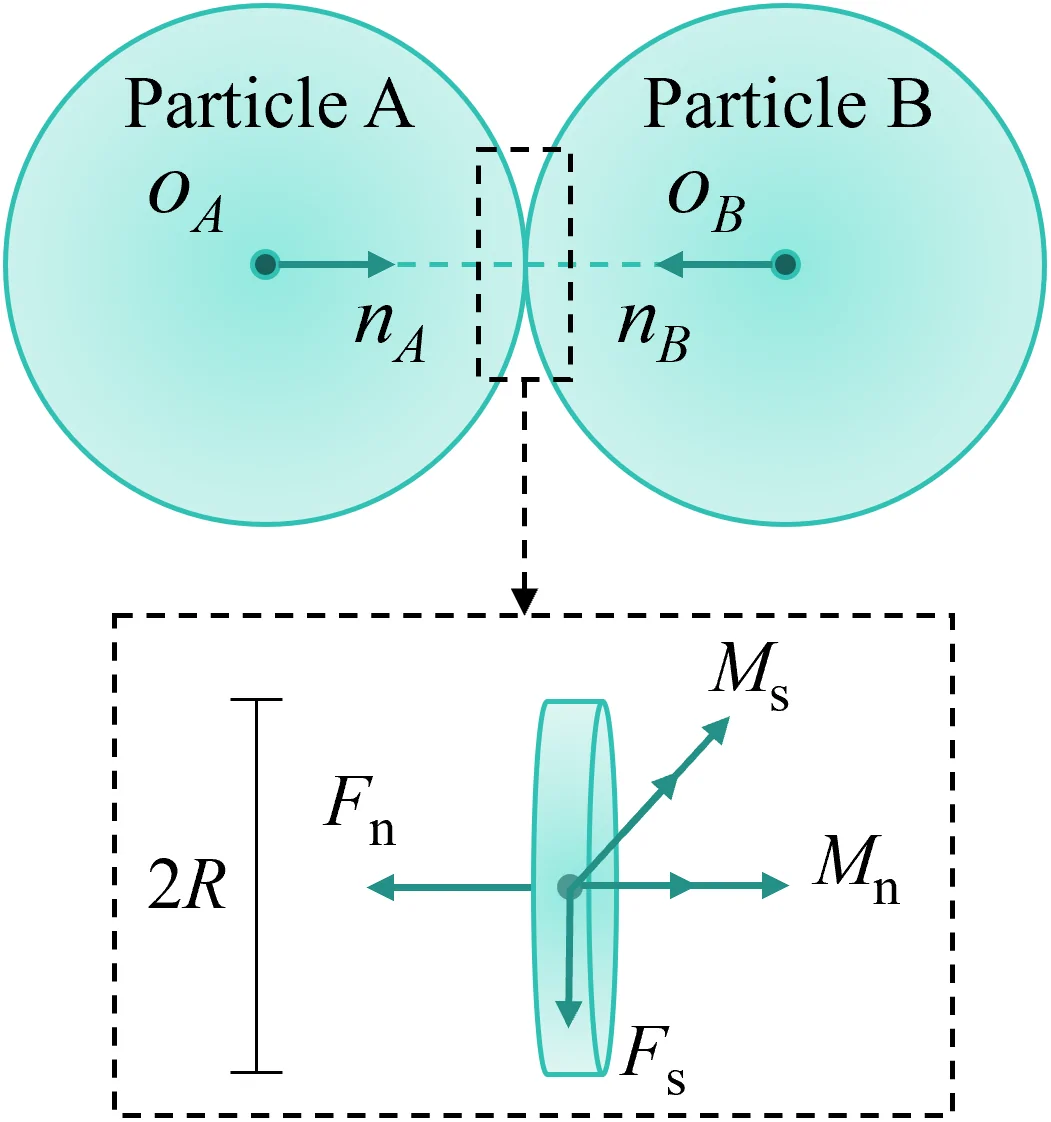
Parallel bonding model between particles.

To achieve the bonding effect between the ballast particles and ice, the parallel bonding model [34] was integrated into the modeling of both the pure ice and the ballast-ice composites. The mechanical effect of this bonding model is to restrict the relative movement between interconnected particles within a specific strength intensity range, thereby establishing a mutually cohesive state. As shown in Fig 8, the calculation domain of the parallel bonding model is characterized as a circular disk with a bonding radius of R formed at the contact interface between two adjacent particles. This spatial configuration enables the bond to transmit not only the forces but also the force moments between particles.

In the parallel bonding model, *F*_n_ is the normal force, *F*_s_ is the tangential force, *M*_n_ is the normal force moment, *M*_s_ is the tangential force moment, and *R* is the radius of disc. The maximum normal stress *σ*_max_ and the maximum shear stress *τ*_max_ acting on the bonded disc can be calculated using Equations (1) and (2). When the maximum normal stress *σ*_max_ is greater than the set normal strength *R*_*n*_ and/or the maximum shear stress *τ*_max_ is greater than the tangential strength *R*_*t*_, the bonding key is damaged, that is, the bonding failure occurs.


$$\begin{array}{c} \sigma_{max}=\frac{-F_{\text{n}}}{A}+\frac{\left|M_{\text{s}}\right|}{I}R \end{array}$$ (1)

$$\begin{array}{c} \tau_{max}=\frac{-F_{\text{s}}}{A}+\frac{\left|M_{\text{n}}\right|}{J}R \end{array}$$ (2)

where *A* is the cross-sectional area of the bonding disc, *I* is the force moment of inertia of the disc’s cross-section, and *J* is the polar moment of inertia. *A*, *I* and *J* can be obtained through Equations (3)–(5).


$$\begin{array}{c} A=\pi R^{\text{2}} \end{array}$$ (3)

$$\begin{array}{c} I=\frac{\text{1}}{\text{4}}\pi R^{\text{4}} \end{array}$$ (4)

$$\begin{array}{c} J=\frac{\text{1}}{\text{2}}\pi R^{\text{4}} \end{array}$$ (5)

#### 3.2.2. DEM models of ballast-ice composites.

**Fig 9:**
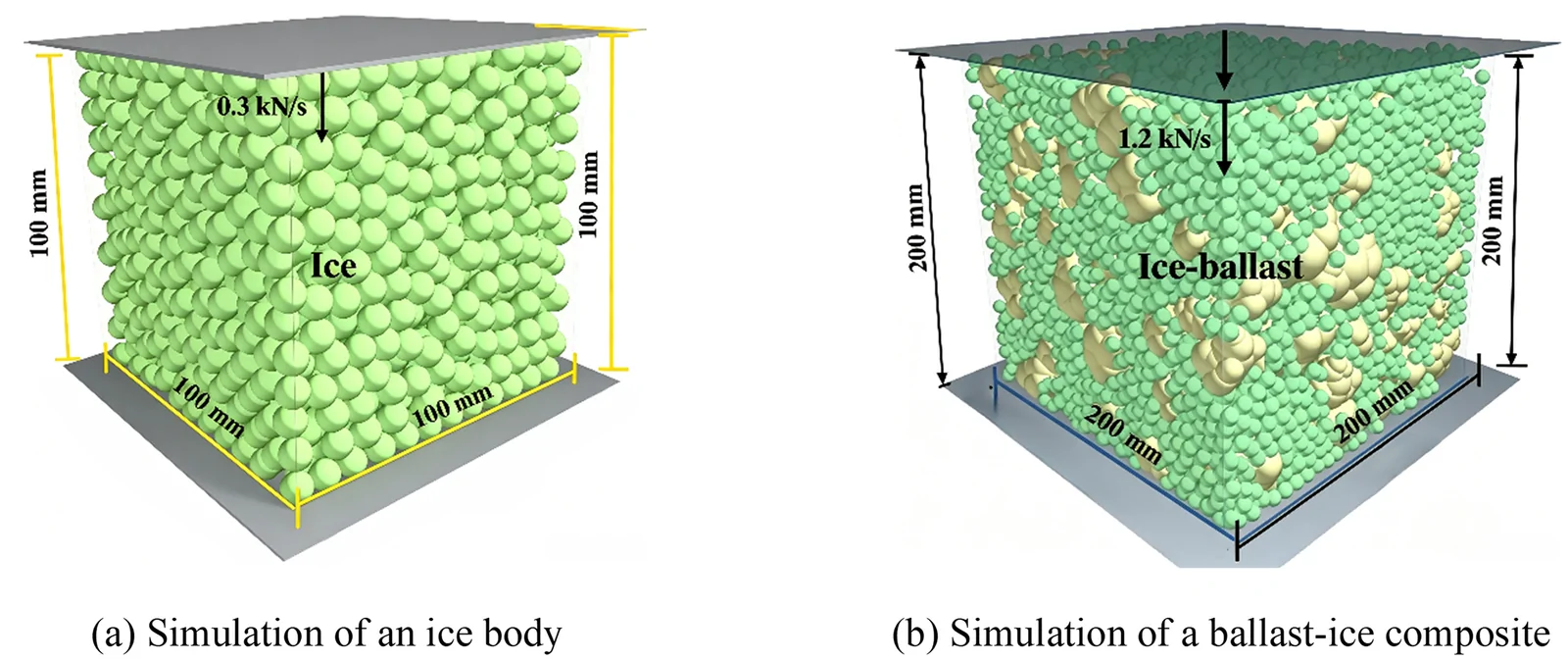
Simulations of an ice body and a ballast-ice composite.

When conducting the modeling and simulations of pure ice using small spherical particles, the radius of the spherical particles was ultimately set to 4 mm, following a comprehensive trade-off between specimen sizes, particle numbers, and computational efficiency. Then, the corresponding DEM models for the uniaxial compression tests of both ice specimens and ballast-ice composites were established. Rigid loading plates with dimensions of 100 mm × 100 mm and 200 mm × 200 mm were loaded at the top of each specimen models. Axial compression was then applied to the specimens at constant force-controlled loading rates of 0.3 kN/s and 1.2 kN/s, respectively, as shown in Fig 9. Through repeated simulation trials and result comparisons, the simulation parameters for the DEM frozen ballast beds were determined, as detailed in Tables 2, 3, 4.

**Table 2:** DEM simulation parameters of ice.

Types	Density	Elastic modulus	Poisson’s ratio	Restitution coefficient	Static friction coefficient	Rolling friction coefficient
**Ice**	920 kg/m^3^	1.20 × 10^9^ Pa	0.30	0.25	0.20	0.01

**Table 3:** DEM simulation parameters of ice bonding keys.

Temperatures	Normal stiffness (×10^8^ N/m^3^)	Shear stiffness (×10^8^ N/m^3^)	Normal strength (×10^6^ Pa)	Shear strength (×10^6^ Pa)	Bonded disk radius (mm)
**−10°C**	10.00	5.00	2.35	1.30	4 ~ 4.5
**−20°C**	12.00	6.00	3.00	1.50	4 ~ 4.5
**−30°C**	14.00	7.00	3.40	1.70	4 ~ 4.5

**Table 4:** DEM simulation parameters of ballast-ice bonding keys.

Temperatures	Normal stiffness (×10^8^ N/m^3^)	Shear stiffness (×10^8^ N/m^3^)	Normal strength (×10^6^ Pa)	Shear strength (×10^6^ Pa)	Bonded disk radius (mm)
**−10°C**	20.00	10.00	5.30	2.60	4 ~ 30
**−20°C**	25.00	12.00	7.00	3.50	4 ~ 30
**−30°C**	25.00	12.50	9.50	4.80	4 ~ 30

## 4. Verifications of DEM models

### 4.1. Verification of ballast bed lateral resistance

**Fig 10:**
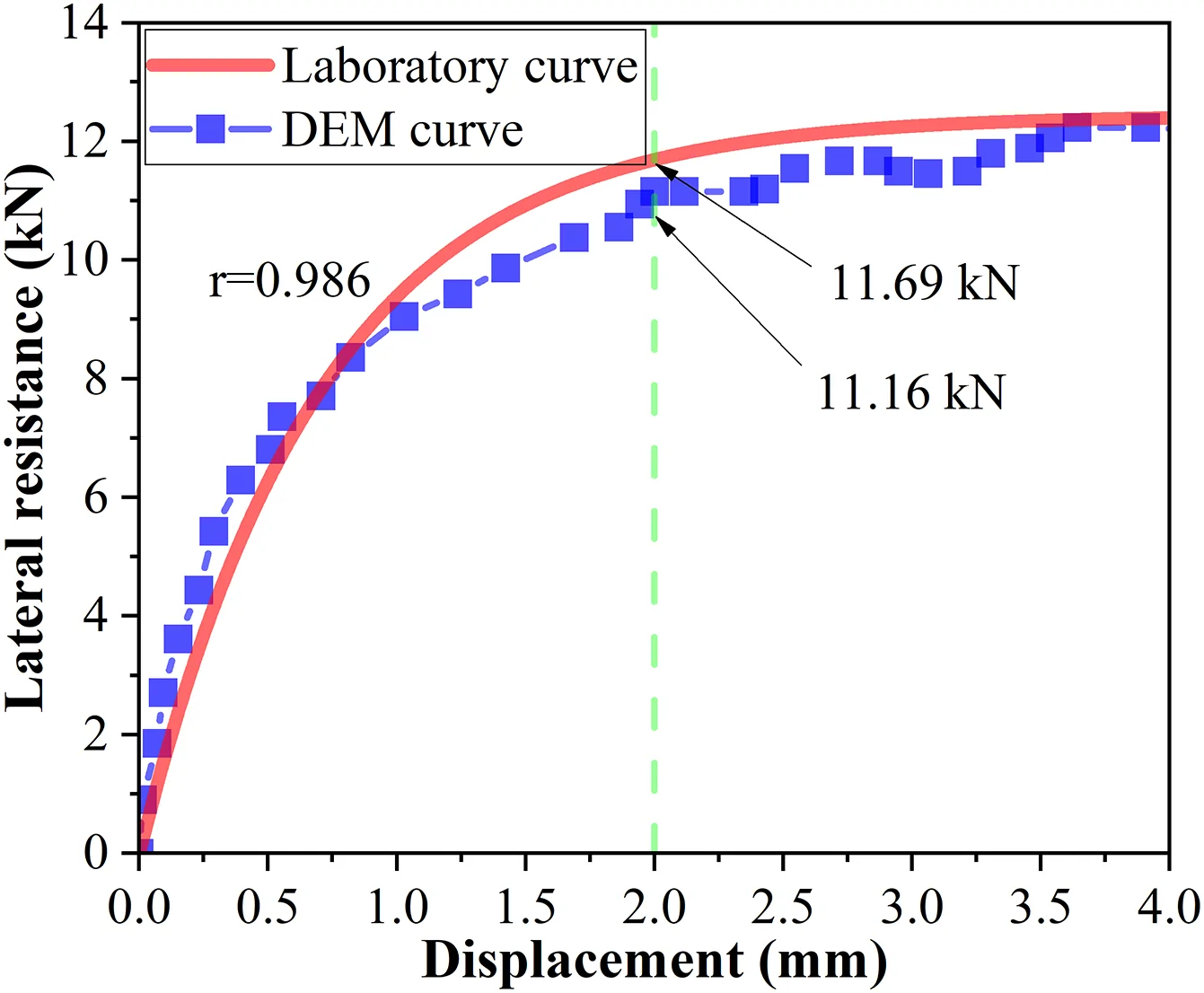
Lateral resistance curves of the laboratory ballast bed and DEM ballast bed.

**Fig 11:**
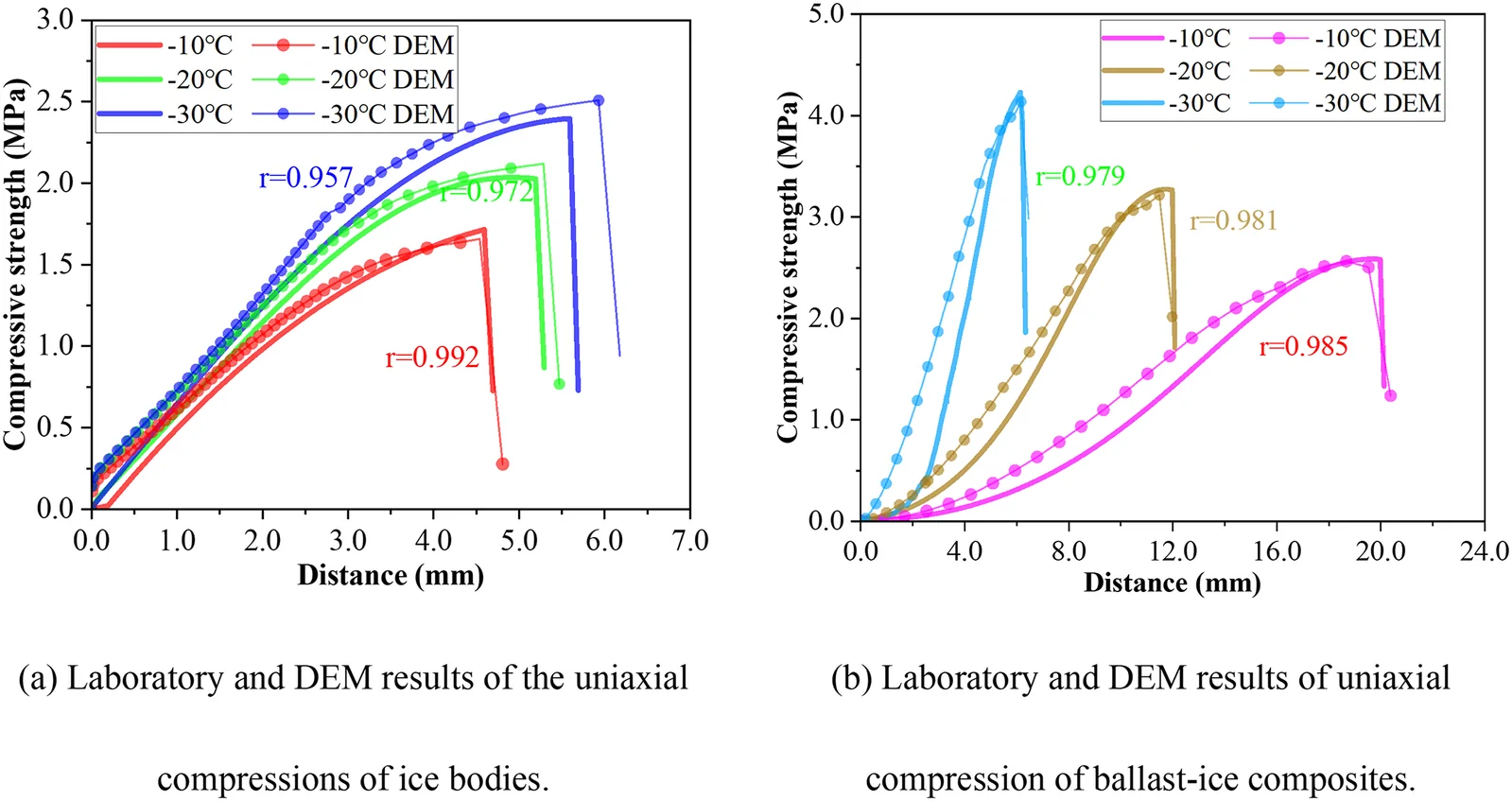
Uniaxial compression curves of ice bodies and ballast-ice composites in the laboratory and DEM under different temperature conditions.

Fig 11 is a comparison of the lateral resistance-displacement curves of the ballast bed obtained from the laboratory tests and the DEM simulations. As observed from Fig 10, the DEM results exhibit a high degree of consistency with the trend of the laboratory results (r = 0.916). Specifically, at a lateral sleeper displacement of 2 mm [35], the laboratory-measured lateral resistance is 11.69 kN, and the corresponding DEM resistance is 11.16 kN. The discrepancy between the two results is merely 4.53%, thereby the applicability of the DEM models for simulating and analyzing the lateral resistance performance of ballast beds is validated.

### 4.2. Laboratory and DEM results of uniaxial compression tests

**Fig 12:**
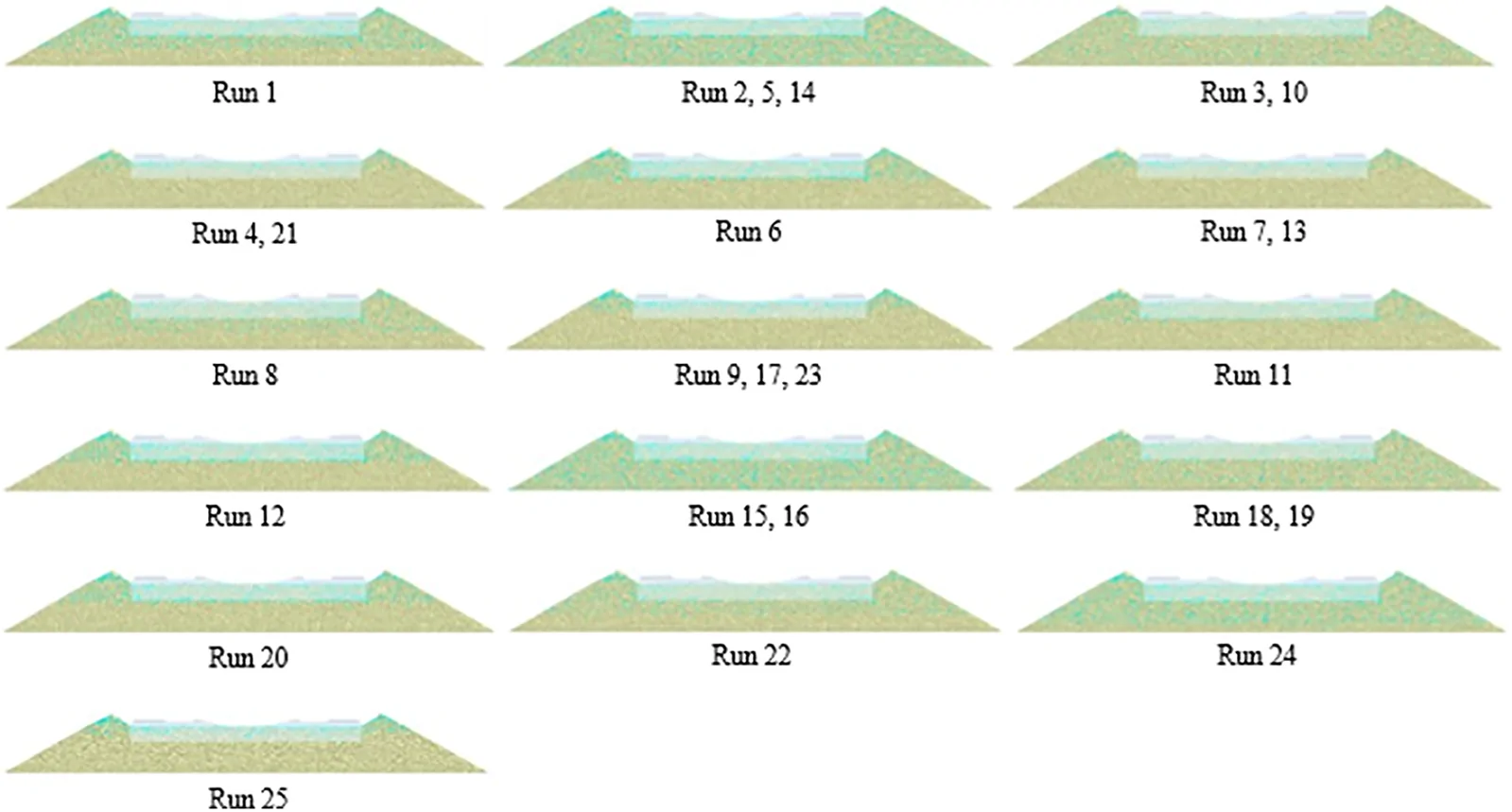
Cross-sections of the DEM ballast beds under different working conditions.

Fig 12 is the laboratory and DEM uniaxial compression curves of ice specimens and ballast-ice composites under various temperature conditions. As shown in Fig 11(a), for the ice specimens, the peak compressive strength increases significantly with decreasing temperature. The peak strength is 1.65 MPa at −10°C, and it rises to 2.48 MPa when the temperature drops to −30°C. The experimental curves show good agreement with the DEM simulations across the initial loading stage, the peak strength point, and the post-peak failure stage, with correlation coefficient r-values of 0.907 (−10°C), 0.923 (−20°C), and 0.955 (−30°C), respectively. This indicates that the DEM models reproduce the mechanical response of ice under low-temperature conditions. Fig 11(b) displays the uniaxial compression behaviors of ballast-ice composites. Compared with the ice specimens, the overall compressive strength of ballast-ice composites is substantially enhanced, with the peak strength reaching approximately 4.50 MPa. Furthermore, the corresponding axial displacement at failure increases markedly, reaching a maximum of around 20 mm, which indicates an enhanced toughness. The DEM curves at all three temperatures are similar with the experimental results, and the R-values are 0.907 (−10°C), 0.917 (−20°C), and 0.955 (−30°C), respectively. These results demonstrate the validity of the established DEM models in capturing the inter-particle bonding mechanisms between ballast aggregates and ice.

The experimental findings indicate that temperature exerts a significant influence on the mechanical characteristics of both ice and ballast-ice composites. The compressive strengths of both materials exhibit a pronounced upward trend with temperature declines. This macroscopic behavior is consistent with the microscopic mechanism wherein the strength of ice crystals increases and the creep effect is attenuated as the temperature decreases [36,37]. It is worth noting that the peak strength of ballast-ice composite is approximately 1.5 to 2.0 times that of ice specimen, while concurrently exhibiting superior ductility characteristics. This phenomenon demonstrates that the ballast particles, through interfacial bonding and skeleton interlocking effects, effectively restrain the brittle failure of ice, thereby substantially enhancing the macroscopic mechanical performance of the ballast-ice composites.

## 5. Analysis of ballast bed resistances under progressive freezing action of ice layers

The infiltration and freezing of ice within the ballast bed is a progressive process. During this process, the degree of freezing among individual ballast particles is predominantly governed by the ice content, freezing temperature, and freeing depth within the ballast layer. Considering the high cost and time-consuming nature of freezing laboratory tests on ballast beds, this chapter utilizes the validated DEM models established in Chapter 3 and Chapter 4 to investigate the macro-mesoscopic influences of ice content, freezing temperature, and freeing depth on the lateral resistance performances of frozen ballast beds.

### 5.1. Working condition design of progressive freezing

Based on the thickness (485 mm) of a newly constructed Class I ballast bed with two 150 mm shoulders [31] and the ice content ranges affecting the mechanical properties of the ballast layer (5% to 15%, 15% to 25%) [24], this section uses the Response Surface Methodology (RSM) to investigate the effects of temperature (−30°C, −20°C, −10°C), ice content (5%, 15%, 25%), and freeing depth (100 mm, 200 mm, 300 mm, 400 mm, 485 mm) on the lateral resistances of frozen ballast beds. The D-optimal design within RSM is selected to generate an efficient experimental matrix, balance experimental costs, and retain statistical power [38].

The core principle of D-optimal design is to maximize the determinant of information matrix, expressed as: max|**X**^T^**X**|. **X** is expanded design matrix, whose columns contain the coded levels (or actual levels) corresponding to regression coefficients, including model intercepts, main effects, and interaction terms. Statistically, this principle is equivalent to minimizing the generalized variance of parameter estimations, which corresponds to minimizing the determinant of coefficient covariance matrix $\text{det}\left(\text{Cov}\left(\hat{\beta }\right)\right)$. Because the coefficient covariance matrix is inversely proportional to the information matrix (that is, $\text{Cov}\left(\hat{\beta }\right)\propto {\left({𝐗}^{\text{T}}𝐗\right)}^{-\text{1}}$), maximizing max|**X**^T^**X**| minimizes the volume of joint confidence ellipsoid of the parameter estimations, thereby providing precise parameter estimates. Thus, the resulting experimental matrix designed for this section is detailed in Table 5.

**Table 5:** Design of test conditions and results.

Run	Factor 1 Temperature (°C)	Factor 2 Ice contents (%)	Factor 3 Depth (mm)	Result Lateral resistance (kN)
**1**	−20	15	300	33195.70
**2**	−10	25	485	39570.20
**3**	−30	5	485	22195.00
**4**	−30	5	100	16115.10
**5**	−10	25	485	39570.20
**6**	−30	15	200	30106.30
**7**	−10	5	100	15346.70
**8**	−10	5	300	19677.00
**9**	−30	25	100	21145.10
**10**	−30	5	485	22195.00
**11**	−20	5	200	19419.30
**12**	−20	25	200	35292.60
**13**	−10	5	100	15346.70
**14**	−10	25	485	23276.60
**15**	−30	25	485	39828.10
**16**	−30	25	485	39828.10
**17**	−30	25	100	21145.10
**18**	−10	5	485	20344.10
**19**	−10	5	485	20344.10
**20**	−10	15	200	30479.00
**21**	−30	5	100	16115.10
**22**	−30	5	300	19976.00
**23**	−10	25	100	19625.30
**24**	−30	15	400	32586.30
**25**	−10	25	100	19625.30

### 5.2. DEM models of ballast bed lateral resistances under progressive freezing action of ice layers

The ice content ω of a ballast bed is defined as follows:


$$\begin{array}{c} \omega =\frac{V_{\text{ice}}}{V_{\text{pore}}} \end{array}$$ (6)

where *V*_ice_ is the volume of ice within ballast bed, which can be calculated as *V*_ice_=(*m*_w_-*m*_b_)/*ρ*_ice_. *m*_w_ is the mass of frozen ballast bed. *m*_b_ is the mass of dry ballast bed, and *ρ*_ice_ is the density of ice (*ρ*_ice_ = 920 kg/m^3^). *V*_pore_ is the total volume of voids in ballast beds under dry condition, computed as *V*_pore_ = *V*-*m*_b_/*ρ*_b_, where *V* is the apparent volume of ballast bed and *ρ*_b_ is the bulk density of ballast bed. Then the parallel bonding model and the obtained calibrated parameters were used. DEM models of frozen ballast beds under various progressive freezing conditions were established based on the three-sleeper ballast bed model shown in Fig 7 and the corresponding simulated scenarios in Table 5, as shown in Fig 12. Upon completion of the DEM simulations, the ballast bed lateral resistance values at the tested sleeper displacements of 2 mm under each working condition were obtained and recorded in the “Result” column of Table 5.

### 5.3. Prediction model of ballast bed lateral resistances under progressive freezing actions

The D-optimal design comprises a total of 25 experimental runs, including replicates for pure error estimation and additional points for the lack-of-fit tests (Runs 5, 10, 13, 14, 16, 17, 19, 21, 23). The design conditions selected in this paper operates under a purely categorical factors mode, meaning there are no continuous axes, and consequently, fitting quadratic terms for independent variables is not required. The fitting approach utilizes the “Main effects+2FI” structure (i.e., Intercept+A + B + C + AB + AC + BC). Therefore, the fitting equation for ballast bed lateral resistance is expressed as follows:


$$\begin{array}{c} F=a\times A+b\times B+c\times C+d\times AB+e\times AC+f\times BC+g \end{array}$$ (7)

where *A* is temperature, *B* is ice content, and C is freezing depth. *a*, *b*, *c*, *d*, *e*, *f* are coefficients of the corresponding terms, and *g* is a constant term.

**Fig 13:**
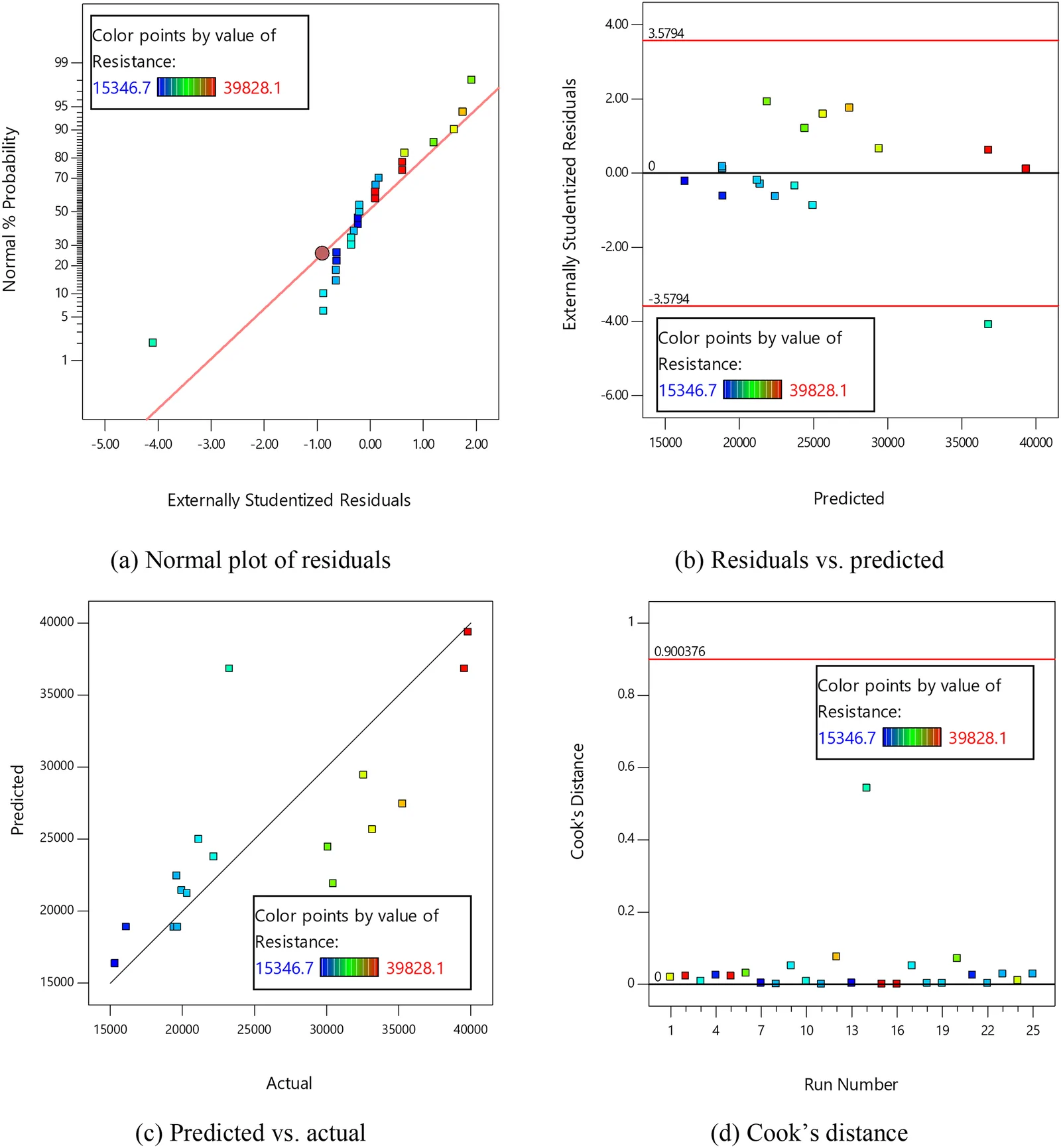
Residual analysis of the model fitting results.


Following stepwise refinement and simplification of the model, a reduced two-factor interaction (2FI) model incorporating the main effects of *A*, *B*, *C*, and the BC interaction term was adopted for analysis. That is, *a* = −127.0766, *b* = 180.6144, *c* = 6.4625, *d* = 0, *e* = 0, *f* = 1.2361, *g* = 12906.0629. The analysis of variance (ANOVA) results indicate that the model is statistically significant as a whole, with an F-value = 12.10 and a p-value less<0.0001. The main effects of ice content (*B*, p-value<0.0001) and freeing depth (*C*, p-value = 0.0006) are highly significant. However, the p-value of temperature (*A*) is 0.2507, indicating that its main effect was not significant. The *BC* interaction term is marginally significant (p-value = 0.0627), indicating that the impact of ice content on lateral resistance depends on the freeing depth. Regarding the goodness-of-fit indicators, the coefficient of determination R^2^ = 0.7076, the Adjusted R^2^ = 0.6491, the Predicted R^2^ is 0.5710, and the Adequate Precision is 10.24, demonstrating that the model has a good predictive ability and can be effectively used for the analysis of relevant patterns. The Variance Inflation Factors (VIF) for *A*, *B*, *C*, *BC* are all approximately 1.01, indicating the absence of multicollinearity among the model terms. The residual analysis of model fitting is shown in Fig 13. The results of Normal plot of residuals, Residuals vs. predicted, Predicted vs. actual, and Cook’s distance indict that the residuals of fitting model meet the assumed conditions such as normality and homogeneity of variance, and no significant influence point is found. The final predictive model for ballast bed lateral resistance under progressive ice freezing action is established, as shown in Equation (8).

$$\begin{array}{c} F=-127.0766A+180.6144B+6.4625C+1.2361BC+12906.0629 \end{array}$$ (8)

### 5.4. Macroscopic laws of frozen ballast bed lateral resistance and parameter sensitivity

Based on the predictive model in Equation (8), a parametric sensitivity analysis was conducted on the evolutionary patterns of ballast bed lateral resistance under ice progressive freezing. As detailed in Section 5.3, the ice content and freezing depth are primary factors governing the lateral resistance, with both main effects being highly significant (p-value<0.001 and p-value = 0.0006, respectively). When the ice content increases from 5% to 25%, the resistance increments range from 50% to over 100% across different freezing depths. Meanwhile, the impact of freezing depth on the resistance exhibits an approximately linear positive correlation. This is attributed to the fact that an increase in ice content leads to a higher number of inter-particle ice bonding keys among ballast aggregates. The relative sliding and rolling between particles are subjected to stricter constraints, resulting in an enhancement of lateral resistance. Furthermore, a greater freezing depth implies a larger volume of frozen ballast mobilized to resist lateral displacement, leading to a more continuous “ballast-ice skeleton” structure and a stronger lateral constraint on the tested sleeper. As the freezing depth increases from 100 mm to 485 mm, the lateral resistance increases by an average of approximately 40% to 80%. The main effect of temperature is not statistically significant (p-value = 0.2507), but its indirect influence is reflected via the *BC* interaction term (p-value = 0.0627, marginally significant). At lower temperatures (e.g., −30°C), the freezing strength of ice is higher (see Tables 3 and Table 4), rendering the bindings between ballast and ice less susceptible to failure under identical ice content and depth conditions, which marginally increases the resistance. This phenomenon is consistent with the evolutionary trend observed in the uniaxial compression tests, where the peak strength increases with decreasing temperature. However, within the temperature range investigated in this study (−10°C to −30°C), the contribution of temperature to macro-resistance is partially masked by the dominant main effects of ice content and freezing depth. The above findings will be further integrated with the mesoscopic analysis in Section 5.5.

**Fig 14:**
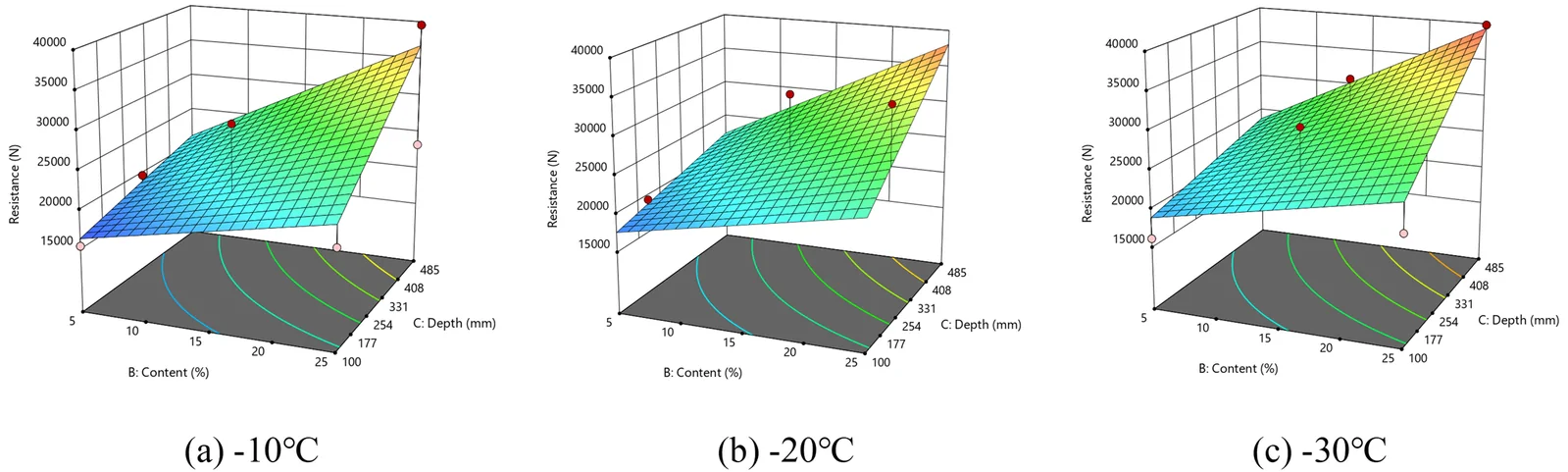
Response surface plots of lateral resistance under different temperatures, ice contents and freezing depths.

**Fig 15:**
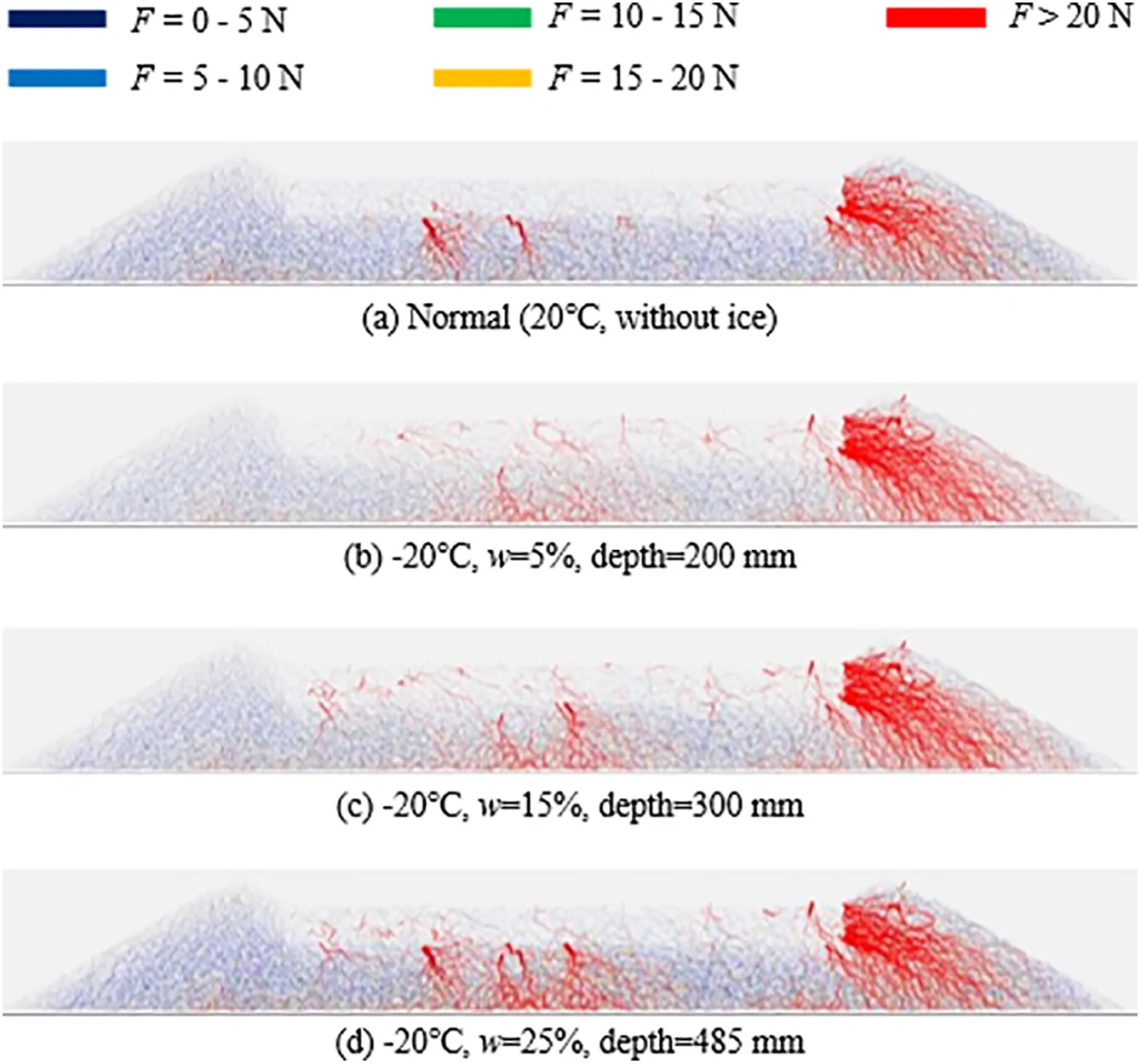
Distributions of contact force chains of ballast beds under different freezing conditions.

To visualize the parametric dependencies, response surface plots showing the interactions among temperature, ice content, and freezing depth were generated based on the predictive model (Fig 14). As observed from Fig 15, the lateral resistance reaches its maximum value (approaching 40 kN) in the high ice content and deep freezing depth region. Conversely, the lateral resistance drops to its minimum (approximately 15 kN) in the low ice content and shallow freezing depth region, which is close to the experimentally measured resistance under conventional un-frozen condition (approximately 11–12 kN). This outcome indicates that the initial stage of ice progressive freezing (characterized by a shallow freezing depth and low ice content) provides limited enhancement to the stability of ballast bed. However, as the freezing process advances, the strengthening effect becomes pronounced. Furthermore, these findings serve as a practical warning for the spring thaw period. The frozen ballast bed lateral resistance can decrease rapidly as ice layers melt, thereby elevating the risk associated with track instability.

### 5.5. Mesoscopic mechanisms of lateral resistance evolution under progressive freezing actions

The analysis in Section 5.4 indicates that the effect of freezing temperature on ballast bed lateral resistance is less significant than that of ice content and freezing depth. To further reveal the mesoscopic mechanisms underlying the evolution of ballast bed lateral resistance under progressive freezing actions, this section selects three representative simulated conditions for comparative analysis, alongside a conventional un-frozen ballast bed (20°C, ice-free). The freezing temperature is set to −20°C, which represents a typical freezing temperature along plateau mountainous railways in China [25]. These three benchmark freezing conditions comprise: low ice content-shallow freezing (5%, 200 mm), moderate ice content-medium freezing (15%, 300 mm), and high ice content-deep freezing (25%, 485 mm). The spatial distribution of contact force chains, particle movements, coordination numbers, and bonding key characteristics are extracted and evaluated. All mesoscopic data are extracted at a lateral sleeper displacement of 2 mm, because the displacement corresponds to the key stage when ballast bed resistance takes effect in real engineering [39,40].

#### 5.5.1. Distribution characteristics of contact force chains.

Fig 15 is the spatial distribution of contact force chains within ballast beds under conventional un-frozen state and the three representative simulated conditions. As shown in Fig 15(a), the overall contact force chains are weak, predominantly consisting of blue and light-blue weak force chains (0–10 N) under un-frozen conditions. A limited number of red strong force chains (>20 N) appear in the frontal zone along the direction of sleeper displacement, in particular, the strong force chain network beneath the tested sleeper bottom is sparse and has an uneven distribution. This phenomenon indicates that the load is transferred primarily through a small number of particle contact points, and the ballast bed exhibits typical characteristics of a loose granular medium. With the occurrence of ice progressive freezing, the force chain distribution alters. In low ice content-shallow freezing condition (Fig 15(b)), weak force chains remain prevalent, yet the quantity and spatial extent of red strong force chains increase, with the strong force chains expanding from the sleeper sides into the interior of ballast layer. This observation demonstrates that the ice bonding mechanism initiates an enhancement in the force transmission capacity among particles. Under the moderate ice content-medium freezing condition (Fig 15(c)), the quantity of strong force chains increases further, elevating the density of the force chain network. The red strong force chains become more concentrated in the frontal zone of sleeper displacement and propagate deeper into ballast bed, with certain force chains displaying arching or chain-like characteristics, thereby diversifying the load transfer paths. When the ballast bed undergoes high ice content-deep freezing (Fig 15(d)), the proportion of strong force chains exhibits a marked increase, resulting in a highly developed and more uniform force chain network. Particularly, owing to the ice bonding effect, the distribution of strong force chains beneath sleeper bottom transitions from being sparse and discrete to uniform and contiguous. Compared with the un-frozen condition, both the quantity and strength of strong force chains within the ballast bed are enhanced, and the configuration of force chain network transforms from locally concentrated and discontinuous to overall dense and stable.

The above phenomena indict that the progressive freezing of ice layer enhances the force transmission capacity among particles by increasing additional bonding keys between ballast particles and ice. The ballast layer undergoes a gradual transition from a friction-dominated loose system to a composite stable system characterized by the co-action of both bonding and friction, thereby providing stronger lateral restriction for sleepers. Furthermore, the increased density and deeper expansion of strong force chain networks effectively suppress particles sliding and rearrangement during lateral sleeper displacements. This structural change is a primary mechanistic factor driving the substantial escalation of ballast bed lateral resistances under middle and high freezing conditions.

#### 5.5.2. Characteristics of particle movements, coordination numbers, and bonding keys.

**Fig 16:**
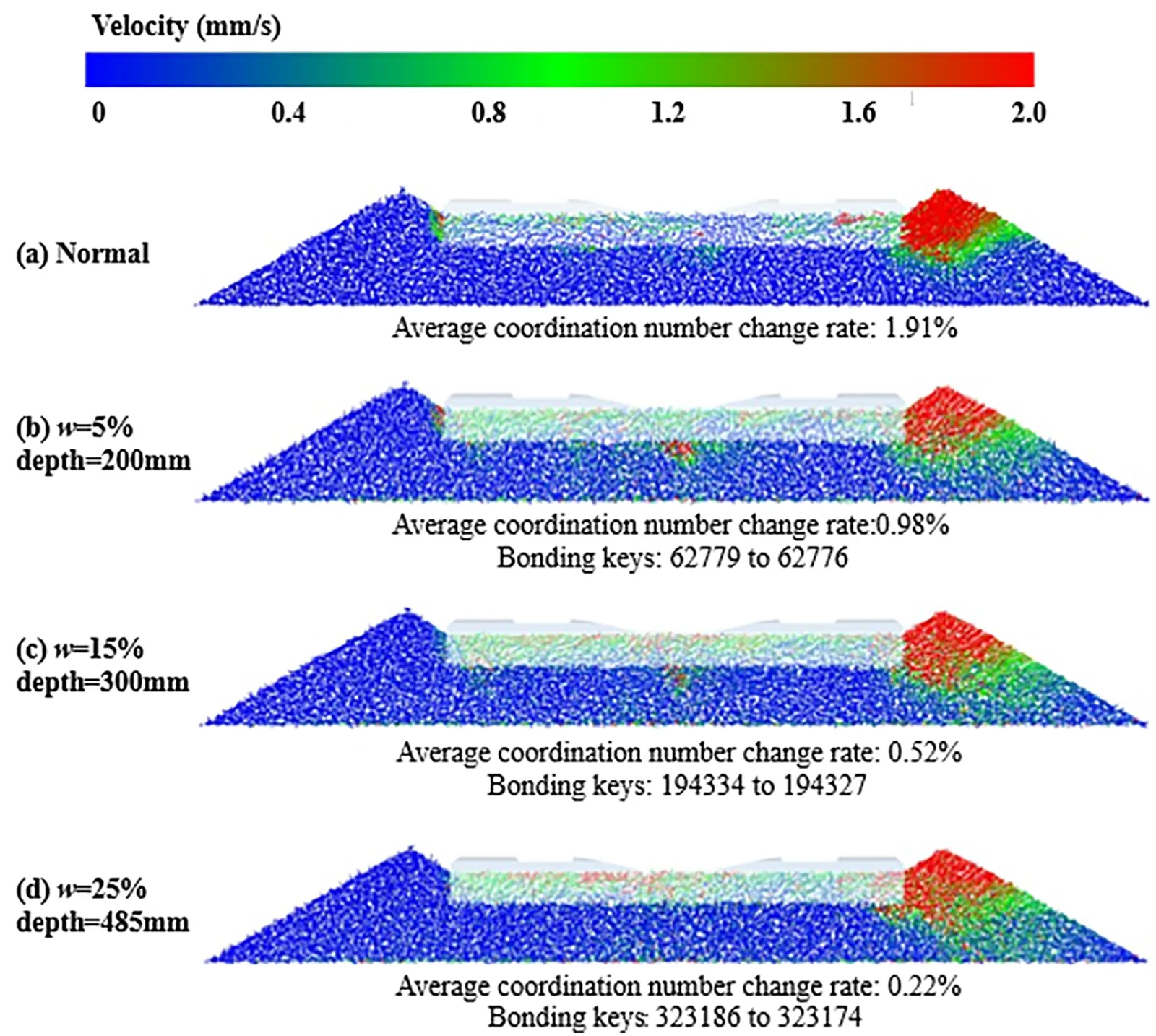
Characteristics of particle movements, coordination numbers and bonding keys in the ballast beds under different freezing conditions.

Fig 16 shows the particle velocity fields, the variations in mean coordination number, and the evolutionary trends of bonding keys under different working conditions. As observed from Fig 16, under same velocities of tested sleepers, the ballast bed in un-frozen state exhibits the most expansive high-velocity zone of particle movement (red vectors with velocities>1.6 mm/s). This zone is primarily concentrated within the frontal area along the direction of tested sleeper displacement and inside the shear band. A large number of particles appear sliding and rearrangement, and the rate of change in the mean coordination number is 1.91%. This indicates that particle interactions within the un-frozen ballast bed is dominated by loose frictional contact, resulting in low resistance to deformation. After the adding and freezing of a minor volume of ice (the low ice content-shallow freezing condition), the extent of high-velocity zone decreases, and the particle movement near the right end of sleeper remains active. This suggests that while the ice bonding mechanism initiates its effect, the restriction remains constrained owing to the shallow freezing depth and low ice content, with the rate of change in the mean coordination number dropping to 0.98%. As the ice content and freezing depth further increase (the moderate ice content-medium freezing condition), the high-velocity regions contract further, with the red zone localized adjacent to the right side of tested sleeper. The rate of change in the mean coordination number is 0.52%, and the bonding keys experience minor broken yet maintain a big number (decreasing from 194334 to 194327), imposing a moderate restriction on particle movements. Concurrently, the number of particles forced to move on the side of sleeper (crib ballast zone) due to the increase of bonding keys, and the velocities of these particles are nearly at low-to-medium levels (indicated in green and blue). In the high ice content-deep freezing condition, the high-velocity zone exhibits the minimum spatial extent and a reduced intensity, confined to a small region at the upper right end of tested sleeper. Most of the particles remain in a low-velocity state, (blue and green regions dominating the velocity field). The rate of change in the mean coordination number minimizes to 0.22%, and the bond count escalates to 323174. The change rate of average coordination number is 0.22%, and the number of bonding keys increases to 323174. This indicates that under the condition of high ice content and deep freezing, many bonding keys between ballast and ice particles form a relatively tight bonding network, restricting the relative movement and spatial distribution of these particles, which causing the ballast bed to exhibit structural “solidification” characteristics.

## 6. Conclusions

The ballast beds of cold-region railways face the impact of progressive ice freezing during operation, leading to complex evolutions of their lateral resistances. To address this problem, this paper studied the evolution patterns and mesoscopic strengthening mechanisms of the ballast bed lateral resistances under progressive ice freezing actions, by means of laboratory lateral resistance tests, uniaxial compression tests, the discrete element method (DEM), and the D-optimal response surface design. The main conclusions are as follows:

(1) Uniaxial compression tests on the ballast-ice composites indicate that the peak compressive strength of a ballast-ice composite increases as temperature decreases (−10°C to −30°C), and the composite exhibits ductility properties superior to those of pure ice.
(2) The DEM models established based on the parallel-bond model can simulate the uniaxial compression behaviors of ballast-ice composites, and together with the DEM models of ballast bed lateral resistances, validates the effectiveness of frozen ballast bed DEM models.
(3) A predictive model for the ballast bed lateral resistance under progressive ice freezing was constructed using the D-optimal design and DEM simulations. Ice content and freezing depth are the dominant factors, both showing a positive correlation. The main effect of temperature is not significant, but it indirectly affects the lateral resistance through interactions.
(4) Progressive ice freezing transforms the ballast bed from a friction-dominated loose system into a bonding-friction composite stable system by increasing the number of bonding keys, increasing the particle coordination number, and strengthening the contact force chain network, thereby increasing the ballast bed lateral resistance.

The above research results can provide a quantitative method and theoretical basis for the operation performance evaluation and maintenance strategy formulation of cold-region railway ballasted tracks. It is worth noting that through the combined conditions of different ice contents, freezing temperatures, and freezing depths, it can be observed that although the freezing effect of ice layers can increase the lateral stability of ballast bed, the melting of the ice layers during temperature transitions may lead to a sharp decline in the ballast bed lateral resistance. In future work, continuous track stiffness monitoring points (e.g., using vehicle-mounted or stationary stiffness detection devices) can be deployed in key sections of cold-region railways to acquire real-time variations in ballast bed support stiffness. When a noticeable decreasing trend in stiffness is detected, the present model can rapidly estimate the current lateral resistance level of the ballast bed by incorporating ambient temperatures and historical freezing data, thereby providing early warnings for frost damage.

## Supporting information

S1 Data Data supplementary information. (DOCX)
