# Supplementary material for: Evolution patterns and characteristics of ballasted track bed lateral resistances in cold regions under progressive freezing of ice layers
Source: PLoS One. 2026 Aug 27;21(8):e0356815. doi: 10.1371/journal.pone.0356815 (PMC13521410; doi:10.1371/journal.pone.0356815)
Supplement: Supplementary file 1 [file pone.0356815.s001.docx]

**Supplementary Information**

**S1 Fig. Fig 1. Particle size distribution. (data of ‘Samples’)**

| Size (mm) | 16 | 25 | 35.5 | 45 | 56 | 63 |
| --- | --- | --- | --- | --- | --- | --- |
| Cummulative passing (%) | 0 | 12 | 32 | 67 | 95 | 100 |

**S2 Fig. Fig 11. Lateral resistance curves of the laboratory ballast bed and DEM ballast bed.**

DEM：

| **No.** | **Displacement (mm)** | **Lateral resistance (kN)** |
| --- | --- | --- |
| 1 | 0.0000 | 0.0000 |
| 2 | 0.0116 | 0.9046 |
| 3 | 0.0694 | 1.8523 |
| 4 | 0.0925 | 2.7138 |
| 5 | 0.1503 | 3.6185 |
| 6 | 0.2312 | 4.4369 |
| 7 | 0.2890 | 5.4277 |
| 8 | 0.3931 | 6.2892 |
| 9 | 0.5087 | 6.8062 |
| 10 | 0.5549 | 7.3662 |
| 11 | 0.7052 | 7.7108 |
| 12 | 0.8208 | 8.3569 |
| 13 | 1.0289 | 9.0462 |
| 14 | 1.2370 | 9.4338 |
| 15 | 1.4220 | 9.8646 |
| 16 | 1.6879 | 10.3815 |
| 17 | 1.8613 | 10.5538 |
| 18 | 1.9422 | 10.9415 |
| 19 | 2.0000 | 11.1569 |
| 20 | 2.1156 | 11.1569 |
| 21 | 2.3468 | 11.1569 |
| 22 | 2.4277 | 11.2000 |
| 23 | 2.5434 | 11.5446 |
| 24 | 2.7168 | 11.6738 |
| 25 | 2.8671 | 11.6738 |
| 26 | 2.9480 | 11.5015 |
| 27 | 3.0636 | 11.4585 |
| 28 | 3.2139 | 11.5015 |
| 29 | 3.3064 | 11.8031 |
| 30 | 3.4566 | 11.8892 |
| 31 | 3.6299 | 12.0615 |
| 32 | 3.7685 | 12.2254 |
| 33 | 3.9188 | 12.2769 |
| 34 | 4.0690 | 12.3631 |
| 35 | 4.2193 | 12.4062 |
| 36 | 4.3584 | 12.4492 |

Laboratory:

| **No.** | **Displacement (mm)** | **Lateral resistance (kN)** |
| --- | --- | --- |
| 1 | 0.00000 | 0.00000 |
| 2 | 0.01160 | 0.20104 |
| 3 | 0.06940 | 1.15538 |
| 4 | 0.09250 | 1.51564 |
| 5 | 0.15030 | 2.36750 |
| 6 | 0.15270 | 2.40135 |
| 7 | 0.15706 | 2.46268 |
| 8 | 0.16142 | 2.52364 |
| 9 | 0.16578 | 2.58422 |
| 10 | 0.17015 | 2.64443 |
| 11 | 0.17451 | 2.70427 |
| 12 | 0.17887 | 2.76375 |
| 13 | 0.18324 | 2.82287 |
| 14 | 0.18760 | 2.88162 |
| 15 | 0.19196 | 2.94002 |
| 16 | 0.19632 | 2.99806 |
| 17 | 0.20069 | 3.05574 |
| 18 | 0.20505 | 3.11308 |
| 19 | 0.20941 | 3.17006 |
| 20 | 0.21378 | 3.22669 |
| 21 | 0.21814 | 3.28298 |
| 22 | 0.22250 | 3.33893 |
| 23 | 0.22686 | 3.39453 |
| 24 | 0.23120 | 3.44946 |
| 25 | 0.23123 | 3.44979 |
| 26 | 0.23559 | 3.50472 |
| 27 | 0.23995 | 3.55931 |
| 28 | 0.24431 | 3.61357 |
| 29 | 0.24868 | 3.66749 |
| 30 | 0.25304 | 3.72109 |
| 31 | 0.25740 | 3.77436 |
| 32 | 0.26177 | 3.82730 |
| 33 | 0.26613 | 3.87992 |
| 34 | 0.27049 | 3.93222 |
| 35 | 0.27485 | 3.98420 |
| 36 | 0.27922 | 4.03586 |
| 37 | 0.28358 | 4.08721 |
| 38 | 0.28794 | 4.13824 |
| 39 | 0.28900 | 4.15057 |
| 40 | 0.29231 | 4.18896 |
| 41 | 0.29667 | 4.23937 |
| 42 | 0.30103 | 4.28948 |
| 43 | 0.30539 | 4.33928 |
| 44 | 0.30976 | 4.38877 |
| 45 | 0.31412 | 4.43796 |
| 46 | 0.31848 | 4.48685 |
| 47 | 0.32284 | 4.53544 |
| 48 | 0.32721 | 4.58374 |
| 49 | 0.33157 | 4.63174 |
| 50 | 0.33593 | 4.67945 |
| 51 | 0.34030 | 4.72686 |
| 52 | 0.34466 | 4.77399 |
| 53 | 0.34902 | 4.82083 |
| 54 | 0.35338 | 4.86738 |
| 55 | 0.35775 | 4.91365 |
| 56 | 0.36211 | 4.95963 |
| 57 | 0.36647 | 5.00534 |
| 58 | 0.37083 | 5.05076 |
| 59 | 0.37520 | 5.09591 |
| 60 | 0.37956 | 5.14078 |
| 61 | 0.38392 | 5.18538 |
| 62 | 0.38829 | 5.22971 |
| 63 | 0.39265 | 5.27376 |
| 64 | 0.39310 | 5.27831 |
| 65 | 0.39701 | 5.31755 |
| 66 | 0.40137 | 5.36107 |
| 67 | 0.40574 | 5.40432 |
| 68 | 0.41010 | 5.44731 |
| 69 | 0.41446 | 5.49004 |
| 70 | 0.41883 | 5.53250 |
| 71 | 0.42319 | 5.57471 |
| 72 | 0.42755 | 5.61666 |
| 73 | 0.43191 | 5.65835 |
| 74 | 0.43628 | 5.69979 |
| 75 | 0.44064 | 5.74097 |
| 76 | 0.44500 | 5.78190 |
| 77 | 0.44936 | 5.82259 |
| 78 | 0.45373 | 5.86302 |
| 79 | 0.45809 | 5.90321 |
| 80 | 0.46245 | 5.94315 |
| 81 | 0.46682 | 5.98285 |
| 82 | 0.47118 | 6.02230 |
| 83 | 0.47554 | 6.06152 |
| 84 | 0.47990 | 6.10049 |
| 85 | 0.48427 | 6.13923 |
| 86 | 0.48863 | 6.17773 |
| 87 | 0.49299 | 6.21600 |
| 88 | 0.49735 | 6.25403 |
| 89 | 0.50172 | 6.29183 |
| 90 | 0.50608 | 6.32940 |
| 91 | 0.50870 | 6.35184 |
| 92 | 0.51044 | 6.36673 |
| 93 | 0.51481 | 6.40385 |
| 94 | 0.51917 | 6.44073 |
| 95 | 0.52353 | 6.47739 |
| 96 | 0.52789 | 6.51382 |
| 97 | 0.53226 | 6.55004 |
| 98 | 0.53662 | 6.58603 |
| 99 | 0.54098 | 6.62180 |
| 100 | 0.54535 | 6.65735 |
| 101 | 0.54971 | 6.69269 |
| 102 | 0.55407 | 6.72781 |
| 103 | 0.55490 | 6.73446 |
| 104 | 0.55843 | 6.76271 |
| 105 | 0.56280 | 6.79741 |
| 106 | 0.56716 | 6.83189 |
| 107 | 0.57152 | 6.86616 |
| 108 | 0.57588 | 6.90022 |
| 109 | 0.58025 | 6.93407 |
| 110 | 0.58461 | 6.96772 |
| 111 | 0.58897 | 7.00116 |
| 112 | 0.59334 | 7.03439 |
| 113 | 0.59770 | 7.06743 |
| 114 | 0.60206 | 7.10026 |
| 115 | 0.60642 | 7.13289 |
| 116 | 0.61079 | 7.16532 |
| 117 | 0.61515 | 7.19756 |
| 118 | 0.61951 | 7.22959 |
| 119 | 0.62388 | 7.26143 |
| 120 | 0.62824 | 7.29308 |
| 121 | 0.63260 | 7.32453 |
| 122 | 0.63696 | 7.35580 |
| 123 | 0.64133 | 7.38687 |
| 124 | 0.64569 | 7.41775 |
| 125 | 0.65005 | 7.44844 |
| 126 | 0.65441 | 7.47894 |
| 127 | 0.65878 | 7.50926 |
| 128 | 0.66314 | 7.53939 |
| 129 | 0.66750 | 7.56934 |
| 130 | 0.67187 | 7.59911 |
| 131 | 0.67623 | 7.62869 |
| 132 | 0.68059 | 7.65810 |
| 133 | 0.68495 | 7.68732 |
| 134 | 0.68932 | 7.71637 |
| 135 | 0.69368 | 7.74524 |
| 136 | 0.69804 | 7.77393 |
| 137 | 0.70240 | 7.80244 |
| 138 | 0.70520 | 7.82062 |
| 139 | 0.70677 | 7.83079 |
| 140 | 0.71113 | 7.85896 |
| 141 | 0.71549 | 7.88695 |
| 142 | 0.71986 | 7.91478 |
| 143 | 0.72422 | 7.94244 |
| 144 | 0.72858 | 7.96992 |
| 145 | 0.73294 | 7.99724 |
| 146 | 0.73731 | 8.02440 |
| 147 | 0.74167 | 8.05138 |
| 148 | 0.74603 | 8.07821 |
| 149 | 0.75040 | 8.10486 |
| 150 | 0.75476 | 8.13136 |
| 151 | 0.75912 | 8.15769 |
| 152 | 0.76348 | 8.18387 |
| 153 | 0.76785 | 8.20988 |
| 154 | 0.77221 | 8.23573 |
| 155 | 0.77657 | 8.26143 |
| 156 | 0.78093 | 8.28697 |
| 157 | 0.78530 | 8.31235 |
| 158 | 0.78966 | 8.33758 |
| 159 | 0.79402 | 8.36266 |
| 160 | 0.79839 | 8.38758 |
| 161 | 0.80275 | 8.41235 |
| 162 | 0.80711 | 8.43696 |
| 163 | 0.81147 | 8.46143 |
| 164 | 0.81584 | 8.48575 |
| 165 | 0.82020 | 8.50992 |
| 166 | 0.82080 | 8.51323 |
| 167 | 0.82456 | 8.53394 |
| 168 | 0.82892 | 8.55781 |
| 169 | 0.83329 | 8.58154 |
| 170 | 0.83765 | 8.60513 |
| 171 | 0.84201 | 8.62857 |
| 172 | 0.84638 | 8.65186 |
| 173 | 0.85074 | 8.67502 |
| 174 | 0.85510 | 8.69803 |
| 175 | 0.85946 | 8.72091 |
| 176 | 0.86383 | 8.74364 |
| 177 | 0.86819 | 8.76623 |
| 178 | 0.87255 | 8.78869 |
| 179 | 0.87692 | 8.81101 |
| 180 | 0.88128 | 8.83319 |
| 181 | 0.88564 | 8.85524 |
| 182 | 0.89000 | 8.87715 |
| 183 | 0.89437 | 8.89893 |
| 184 | 0.89873 | 8.92058 |
| 185 | 0.90309 | 8.94209 |
| 186 | 0.90745 | 8.96347 |
| 187 | 0.91182 | 8.98472 |
| 188 | 0.91618 | 9.00585 |
| 189 | 0.92054 | 9.02684 |
| 190 | 0.92491 | 9.04770 |
| 191 | 0.92927 | 9.06844 |
| 192 | 0.93363 | 9.08905 |
| 193 | 0.93799 | 9.10954 |
| 194 | 0.94236 | 9.12990 |
| 195 | 0.94672 | 9.15013 |
| 196 | 0.95108 | 9.17024 |
| 197 | 0.95545 | 9.19023 |
| 198 | 0.95981 | 9.21010 |
| 199 | 0.96417 | 9.22984 |
| 200 | 0.96853 | 9.24947 |
| 201 | 0.97290 | 9.26897 |
| 202 | 0.97726 | 9.28836 |
| 203 | 0.98162 | 9.30763 |
| 204 | 0.98598 | 9.32678 |
| 205 | 0.99035 | 9.34581 |
| 206 | 0.99471 | 9.36473 |
| 207 | 0.99907 | 9.38353 |
| 208 | 1.00344 | 9.40221 |
| 209 | 1.00780 | 9.42078 |
| 210 | 1.01216 | 9.43924 |
| 211 | 1.01652 | 9.45759 |
| 212 | 1.02089 | 9.47582 |
| 213 | 1.02525 | 9.49395 |
| 214 | 1.02890 | 9.50903 |
| 215 | 1.02961 | 9.51196 |
| 216 | 1.03397 | 9.52986 |
| 217 | 1.03834 | 9.54765 |
| 218 | 1.04270 | 9.56534 |
| 219 | 1.04706 | 9.58291 |
| 220 | 1.05143 | 9.60038 |
| 221 | 1.05579 | 9.61774 |
| 222 | 1.06015 | 9.63500 |
| 223 | 1.06451 | 9.65215 |
| 224 | 1.06888 | 9.66919 |
| 225 | 1.07324 | 9.68614 |
| 226 | 1.07760 | 9.70297 |
| 227 | 1.08197 | 9.71971 |
| 228 | 1.08633 | 9.73634 |
| 229 | 1.09069 | 9.75287 |
| 230 | 1.09505 | 9.76930 |
| 231 | 1.09942 | 9.78563 |
| 232 | 1.10378 | 9.80186 |
| 233 | 1.10814 | 9.81800 |
| 234 | 1.11250 | 9.83403 |
| 235 | 1.11687 | 9.84996 |
| 236 | 1.12123 | 9.86580 |
| 237 | 1.12559 | 9.88154 |
| 238 | 1.12996 | 9.89719 |
| 239 | 1.13432 | 9.91274 |
| 240 | 1.13868 | 9.92819 |
| 241 | 1.14304 | 9.94355 |
| 242 | 1.14741 | 9.95882 |
| 243 | 1.15177 | 9.97399 |
| 244 | 1.15613 | 9.98907 |
| 245 | 1.16049 | 10.00406 |
| 246 | 1.16486 | 10.01895 |
| 247 | 1.16922 | 10.03376 |
| 248 | 1.17358 | 10.04847 |
| 249 | 1.17795 | 10.06310 |
| 250 | 1.18231 | 10.07763 |
| 251 | 1.18667 | 10.09208 |
| 252 | 1.19103 | 10.10644 |
| 253 | 1.19540 | 10.12071 |
| 254 | 1.19976 | 10.13489 |
| 255 | 1.20412 | 10.14899 |
| 256 | 1.20849 | 10.16300 |
| 257 | 1.21285 | 10.17693 |
| 258 | 1.21721 | 10.19077 |
| 259 | 1.22157 | 10.20453 |
| 260 | 1.22594 | 10.21820 |
| 261 | 1.23030 | 10.23179 |
| 262 | 1.23466 | 10.24529 |
| 263 | 1.23700 | 10.25250 |
| 264 | 1.23902 | 10.25871 |
| 265 | 1.24339 | 10.27206 |
| 266 | 1.24775 | 10.28532 |
| 267 | 1.25211 | 10.29849 |
| 268 | 1.25648 | 10.31159 |
| 269 | 1.26084 | 10.32461 |
| 270 | 1.26520 | 10.33755 |
| 271 | 1.26956 | 10.35041 |
| 272 | 1.27393 | 10.36319 |
| 273 | 1.27829 | 10.37589 |
| 274 | 1.28265 | 10.38852 |
| 275 | 1.28702 | 10.40107 |
| 276 | 1.29138 | 10.41354 |
| 277 | 1.29574 | 10.42593 |
| 278 | 1.30010 | 10.43825 |
| 279 | 1.30447 | 10.45050 |
| 280 | 1.30883 | 10.46267 |
| 281 | 1.31319 | 10.47476 |
| 282 | 1.31755 | 10.48679 |
| 283 | 1.32192 | 10.49873 |
| 284 | 1.32628 | 10.51061 |
| 285 | 1.33064 | 10.52241 |
| 286 | 1.33501 | 10.53414 |
| 287 | 1.33937 | 10.54580 |
| 288 | 1.34373 | 10.55739 |
| 289 | 1.34809 | 10.56891 |
| 290 | 1.35246 | 10.58035 |
| 291 | 1.35682 | 10.59173 |
| 292 | 1.36118 | 10.60304 |
| 293 | 1.36554 | 10.61427 |
| 294 | 1.36991 | 10.62544 |
| 295 | 1.37427 | 10.63655 |
| 296 | 1.37863 | 10.64758 |
| 297 | 1.38300 | 10.65855 |
| 298 | 1.38736 | 10.66944 |
| 299 | 1.39172 | 10.68028 |
| 300 | 1.39608 | 10.69104 |
| 301 | 1.40045 | 10.70174 |
| 302 | 1.40481 | 10.71238 |
| 303 | 1.40917 | 10.72295 |
| 304 | 1.41354 | 10.73346 |
| 305 | 1.41790 | 10.74390 |
| 306 | 1.42200 | 10.75366 |
| 307 | 1.42226 | 10.75428 |
| 308 | 1.42662 | 10.76459 |
| 309 | 1.43099 | 10.77484 |
| 310 | 1.43535 | 10.78503 |
| 311 | 1.43971 | 10.79516 |
| 312 | 1.44407 | 10.80522 |
| 313 | 1.44844 | 10.81523 |
| 314 | 1.45280 | 10.82517 |
| 315 | 1.45716 | 10.83505 |
| 316 | 1.46153 | 10.84487 |
| 317 | 1.46589 | 10.85463 |
| 318 | 1.47025 | 10.86433 |
| 319 | 1.47461 | 10.87398 |
| 320 | 1.47898 | 10.88356 |
| 321 | 1.48334 | 10.89308 |
| 322 | 1.48770 | 10.90255 |
| 323 | 1.49206 | 10.91196 |
| 324 | 1.49643 | 10.92131 |
| 325 | 1.50079 | 10.93061 |
| 326 | 1.50515 | 10.93984 |
| 327 | 1.50952 | 10.94902 |
| 328 | 1.51388 | 10.95815 |
| 329 | 1.51824 | 10.96722 |
| 330 | 1.52260 | 10.97623 |
| 331 | 1.52697 | 10.98519 |
| 332 | 1.53133 | 10.99410 |
| 333 | 1.53569 | 11.00295 |
| 334 | 1.54006 | 11.01174 |
| 335 | 1.54442 | 11.02048 |
| 336 | 1.54878 | 11.02917 |
| 337 | 1.55314 | 11.03781 |
| 338 | 1.55751 | 11.04639 |
| 339 | 1.56187 | 11.05492 |
| 340 | 1.56623 | 11.06340 |
| 341 | 1.57059 | 11.07183 |
| 342 | 1.57496 | 11.08020 |
| 343 | 1.57932 | 11.08853 |
| 344 | 1.58368 | 11.09680 |
| 345 | 1.58805 | 11.10502 |
| 346 | 1.59241 | 11.11319 |
| 347 | 1.59677 | 11.12132 |
| 348 | 1.60113 | 11.12939 |
| 349 | 1.60550 | 11.13741 |
| 350 | 1.60986 | 11.14539 |
| 351 | 1.61422 | 11.15331 |
| 352 | 1.61858 | 11.16119 |
| 353 | 1.62295 | 11.16902 |
| 354 | 1.62731 | 11.17680 |
| 355 | 1.63167 | 11.18453 |
| 356 | 1.63604 | 11.19222 |
| 357 | 1.64040 | 11.19986 |
| 358 | 1.64476 | 11.20745 |
| 359 | 1.64912 | 11.21500 |
| 360 | 1.65349 | 11.22250 |
| 361 | 1.65785 | 11.22996 |
| 362 | 1.66221 | 11.23737 |
| 363 | 1.66658 | 11.24473 |
| 364 | 1.67094 | 11.25205 |
| 365 | 1.67530 | 11.25932 |
| 366 | 1.67966 | 11.26655 |
| 367 | 1.68403 | 11.27374 |
| 368 | 1.68790 | 11.28008 |
| 369 | 1.68839 | 11.28088 |
| 370 | 1.69275 | 11.28798 |
| 371 | 1.69711 | 11.29503 |
| 372 | 1.70148 | 11.30205 |
| 373 | 1.70584 | 11.30902 |
| 374 | 1.71020 | 11.31594 |
| 375 | 1.71457 | 11.32283 |
| 376 | 1.71893 | 11.32967 |
| 377 | 1.72329 | 11.33647 |
| 378 | 1.72765 | 11.34323 |
| 379 | 1.73202 | 11.34995 |
| 380 | 1.73638 | 11.35662 |
| 381 | 1.74074 | 11.36326 |
| 382 | 1.74511 | 11.36985 |
| 383 | 1.74947 | 11.37641 |
| 384 | 1.75383 | 11.38292 |
| 385 | 1.75819 | 11.38940 |
| 386 | 1.76256 | 11.39583 |
| 387 | 1.76692 | 11.40223 |
| 388 | 1.77128 | 11.40859 |
| 389 | 1.77564 | 11.41491 |
| 390 | 1.78001 | 11.42119 |
| 391 | 1.78437 | 11.42743 |
| 392 | 1.78873 | 11.43363 |
| 393 | 1.79310 | 11.43980 |
| 394 | 1.79746 | 11.44592 |
| 395 | 1.80182 | 11.45201 |
| 396 | 1.80618 | 11.45807 |
| 397 | 1.81055 | 11.46408 |
| 398 | 1.81491 | 11.47006 |
| 399 | 1.81927 | 11.47601 |
| 400 | 1.82363 | 11.48191 |
| 401 | 1.82800 | 11.48778 |
| 402 | 1.83236 | 11.49362 |
| 403 | 1.83672 | 11.49942 |
| 404 | 1.84109 | 11.50518 |
| 405 | 1.84545 | 11.51091 |
| 406 | 1.84981 | 11.51660 |
| 407 | 1.85417 | 11.52226 |
| 408 | 1.85854 | 11.52788 |
| 409 | 1.86130 | 11.53143 |
| 410 | 1.86290 | 11.53347 |
| 411 | 1.86726 | 11.53903 |
| 412 | 1.87163 | 11.54455 |
| 413 | 1.87599 | 11.55004 |
| 414 | 1.88035 | 11.55549 |
| 415 | 1.88471 | 11.56092 |
| 416 | 1.88908 | 11.56630 |
| 417 | 1.89344 | 11.57166 |
| 418 | 1.89780 | 11.57698 |
| 419 | 1.90216 | 11.58227 |
| 420 | 1.90653 | 11.58753 |
| 421 | 1.91089 | 11.59275 |
| 422 | 1.91525 | 11.59795 |
| 423 | 1.91962 | 11.60311 |
| 424 | 1.92398 | 11.60824 |
| 425 | 1.92834 | 11.61334 |
| 426 | 1.93270 | 11.61841 |
| 427 | 1.93707 | 11.62344 |
| 428 | 1.94143 | 11.62845 |
| 429 | 1.94220 | 11.62933 |
| 430 | 1.94579 | 11.63343 |
| 431 | 1.95015 | 11.63837 |
| 432 | 1.95452 | 11.64329 |
| 433 | 1.95888 | 11.64817 |
| 434 | 1.96324 | 11.65303 |
| 435 | 1.96761 | 11.65785 |
| 436 | 1.97197 | 11.66265 |
| 437 | 1.97633 | 11.66741 |
| 438 | 1.98069 | 11.67215 |
| 439 | 1.98506 | 11.67686 |
| 440 | 1.98942 | 11.68154 |
| 441 | 1.99378 | 11.68619 |
| 442 | 1.99815 | 11.69081 |
| 443 | 2.00000 | 11.69277 |
| 444 | 2.00251 | 11.69541 |
| 445 | 2.00687 | 11.69998 |
| 446 | 2.01123 | 11.70451 |
| 447 | 2.01560 | 11.70903 |
| 448 | 2.01996 | 11.71351 |
| 449 | 2.02432 | 11.71797 |
| 450 | 2.02868 | 11.72239 |
| 451 | 2.03305 | 11.72680 |
| 452 | 2.03741 | 11.73117 |
| 453 | 2.04177 | 11.73552 |
| 454 | 2.04614 | 11.73984 |
| 455 | 2.05050 | 11.74414 |
| 456 | 2.05486 | 11.74840 |
| 457 | 2.05922 | 11.75265 |
| 458 | 2.06359 | 11.75686 |
| 459 | 2.06795 | 11.76106 |
| 460 | 2.07231 | 11.76522 |
| 461 | 2.07668 | 11.76936 |
| 462 | 2.08104 | 11.77348 |
| 463 | 2.08540 | 11.77757 |
| 464 | 2.08976 | 11.78163 |
| 465 | 2.09413 | 11.78567 |
| 466 | 2.09849 | 11.78969 |
| 467 | 2.10285 | 11.79368 |
| 468 | 2.10721 | 11.79764 |
| 469 | 2.11158 | 11.80159 |
| 470 | 2.11560 | 11.80520 |
| 471 | 2.11594 | 11.80551 |
| 472 | 2.12030 | 11.80940 |
| 473 | 2.12467 | 11.81327 |
| 474 | 2.12903 | 11.81712 |
| 475 | 2.13339 | 11.82094 |
| 476 | 2.13775 | 11.82474 |
| 477 | 2.14212 | 11.82852 |
| 478 | 2.14648 | 11.83227 |
| 479 | 2.15084 | 11.83600 |
| 480 | 2.15520 | 11.83971 |
| 481 | 2.15957 | 11.84339 |
| 482 | 2.16393 | 11.84706 |
| 483 | 2.16829 | 11.85070 |
| 484 | 2.17266 | 11.85432 |
| 485 | 2.17702 | 11.85791 |
| 486 | 2.18138 | 11.86149 |
| 487 | 2.18574 | 11.86504 |
| 488 | 2.19011 | 11.86857 |
| 489 | 2.19447 | 11.87208 |
| 490 | 2.19883 | 11.87557 |
| 491 | 2.20320 | 11.87903 |
| 492 | 2.20756 | 11.88248 |
| 493 | 2.21192 | 11.88590 |
| 494 | 2.21628 | 11.88930 |
| 495 | 2.22065 | 11.89269 |
| 496 | 2.22501 | 11.89605 |
| 497 | 2.22937 | 11.89939 |
| 498 | 2.23373 | 11.90271 |
| 499 | 2.23810 | 11.90601 |
| 500 | 2.24246 | 11.90929 |
| 501 | 2.24682 | 11.91255 |
| 502 | 2.25119 | 11.91579 |
| 503 | 2.25555 | 11.91901 |
| 504 | 2.25991 | 11.92221 |
| 505 | 2.26427 | 11.92540 |
| 506 | 2.26864 | 11.92856 |
| 507 | 2.27300 | 11.93170 |
| 508 | 2.27736 | 11.93482 |
| 509 | 2.28172 | 11.93793 |
| 510 | 2.28609 | 11.94101 |
| 511 | 2.29045 | 11.94408 |
| 512 | 2.29481 | 11.94713 |
| 513 | 2.29918 | 11.95016 |
| 514 | 2.30354 | 11.95317 |
| 515 | 2.30790 | 11.95616 |
| 516 | 2.31226 | 11.95914 |
| 517 | 2.31663 | 11.96209 |
| 518 | 2.32099 | 11.96503 |
| 519 | 2.32535 | 11.96795 |
| 520 | 2.32972 | 11.97085 |
| 521 | 2.33408 | 11.97374 |
| 522 | 2.33844 | 11.97660 |
| 523 | 2.34280 | 11.97945 |
| 524 | 2.34680 | 11.98205 |
| 525 | 2.34717 | 11.98228 |
| 526 | 2.35153 | 11.98510 |
| 527 | 2.35589 | 11.98790 |
| 528 | 2.36025 | 11.99068 |
| 529 | 2.36462 | 11.99344 |
| 530 | 2.36898 | 11.99619 |
| 531 | 2.37334 | 11.99892 |
| 532 | 2.37771 | 12.00163 |
| 533 | 2.38207 | 12.00432 |
| 534 | 2.38643 | 12.00700 |
| 535 | 2.39079 | 12.00967 |
| 536 | 2.39516 | 12.01232 |
| 537 | 2.39952 | 12.01495 |
| 538 | 2.40388 | 12.01756 |
| 539 | 2.40825 | 12.02016 |
| 540 | 2.41261 | 12.02274 |
| 541 | 2.41697 | 12.02531 |
| 542 | 2.42133 | 12.02786 |
| 543 | 2.42570 | 12.03040 |
| 544 | 2.42770 | 12.03156 |
| 545 | 2.43006 | 12.03292 |
| 546 | 2.43442 | 12.03543 |
| 547 | 2.43878 | 12.03792 |
| 548 | 2.44315 | 12.04039 |
| 549 | 2.44751 | 12.04285 |
| 550 | 2.45187 | 12.04530 |
| 551 | 2.45624 | 12.04772 |
| 552 | 2.46060 | 12.05014 |
| 553 | 2.46496 | 12.05254 |
| 554 | 2.46932 | 12.05493 |
| 555 | 2.47369 | 12.05730 |
| 556 | 2.47805 | 12.05965 |
| 557 | 2.48241 | 12.06199 |
| 558 | 2.48677 | 12.06432 |
| 559 | 2.49114 | 12.06664 |
| 560 | 2.49550 | 12.06894 |
| 561 | 2.49986 | 12.07122 |
| 562 | 2.50423 | 12.07349 |
| 563 | 2.50859 | 12.07575 |
| 564 | 2.51295 | 12.07799 |
| 565 | 2.51731 | 12.08022 |
| 566 | 2.52168 | 12.08244 |
| 567 | 2.52604 | 12.08464 |
| 568 | 2.53040 | 12.08683 |
| 569 | 2.53477 | 12.08901 |
| 570 | 2.53913 | 12.09117 |
| 571 | 2.54340 | 12.09328 |
| 572 | 2.54349 | 12.09332 |
| 573 | 2.54785 | 12.09546 |
| 574 | 2.55222 | 12.09758 |
| 575 | 2.55658 | 12.09969 |
| 576 | 2.56094 | 12.10179 |
| 577 | 2.56530 | 12.10387 |
| 578 | 2.56967 | 12.10595 |
| 579 | 2.57403 | 12.10800 |
| 580 | 2.57839 | 12.11005 |
| 581 | 2.58276 | 12.11209 |
| 582 | 2.58712 | 12.11411 |
| 583 | 2.59148 | 12.11612 |
| 584 | 2.59584 | 12.11811 |
| 585 | 2.60021 | 12.12010 |
| 586 | 2.60457 | 12.12207 |
| 587 | 2.60893 | 12.12403 |
| 588 | 2.61329 | 12.12598 |
| 589 | 2.61766 | 12.12792 |
| 590 | 2.62202 | 12.12984 |
| 591 | 2.62638 | 12.13176 |
| 592 | 2.63075 | 12.13366 |
| 593 | 2.63511 | 12.13555 |
| 594 | 2.63947 | 12.13743 |
| 595 | 2.64383 | 12.13929 |
| 596 | 2.64820 | 12.14115 |
| 597 | 2.65256 | 12.14299 |
| 598 | 2.65692 | 12.14483 |
| 599 | 2.66129 | 12.14665 |
| 600 | 2.66565 | 12.14846 |
| 601 | 2.67001 | 12.15026 |
| 602 | 2.67437 | 12.15205 |
| 603 | 2.67874 | 12.15383 |
| 604 | 2.68310 | 12.15559 |
| 605 | 2.68746 | 12.15735 |
| 606 | 2.69182 | 12.15909 |
| 607 | 2.69619 | 12.16083 |
| 608 | 2.70055 | 12.16255 |
| 609 | 2.70491 | 12.16427 |
| 610 | 2.70928 | 12.16597 |
| 611 | 2.71364 | 12.16766 |
| 612 | 2.71680 | 12.16888 |
| 613 | 2.71800 | 12.16935 |
| 614 | 2.72236 | 12.17102 |
| 615 | 2.72673 | 12.17268 |
| 616 | 2.73109 | 12.17433 |
| 617 | 2.73545 | 12.17597 |
| 618 | 2.73982 | 12.17760 |
| 619 | 2.74418 | 12.17923 |
| 620 | 2.74854 | 12.18084 |
| 621 | 2.75290 | 12.18244 |
| 622 | 2.75727 | 12.18403 |
| 623 | 2.76163 | 12.18561 |
| 624 | 2.76599 | 12.18719 |
| 625 | 2.77035 | 12.18875 |
| 626 | 2.77472 | 12.19030 |
| 627 | 2.77908 | 12.19185 |
| 628 | 2.78344 | 12.19338 |
| 629 | 2.78781 | 12.19491 |
| 630 | 2.79217 | 12.19642 |
| 631 | 2.79653 | 12.19793 |
| 632 | 2.80089 | 12.19943 |
| 633 | 2.80526 | 12.20092 |
| 634 | 2.80962 | 12.20240 |
| 635 | 2.81398 | 12.20387 |
| 636 | 2.81834 | 12.20533 |
| 637 | 2.82271 | 12.20678 |
| 638 | 2.82707 | 12.20822 |
| 639 | 2.83143 | 12.20966 |
| 640 | 2.83580 | 12.21108 |
| 641 | 2.84016 | 12.21250 |
| 642 | 2.84452 | 12.21391 |
| 643 | 2.84888 | 12.21531 |
| 644 | 2.85325 | 12.21670 |
| 645 | 2.85761 | 12.21808 |
| 646 | 2.86197 | 12.21946 |
| 647 | 2.86634 | 12.22082 |
| 648 | 2.86710 | 12.22106 |
| 649 | 2.87070 | 12.22218 |
| 650 | 2.87506 | 12.22353 |
| 651 | 2.87942 | 12.22487 |
| 652 | 2.88379 | 12.22621 |
| 653 | 2.88815 | 12.22753 |
| 654 | 2.89251 | 12.22885 |
| 655 | 2.89687 | 12.23016 |
| 656 | 2.90124 | 12.23146 |
| 657 | 2.90560 | 12.23275 |
| 658 | 2.90996 | 12.23403 |
| 659 | 2.91433 | 12.23531 |
| 660 | 2.91869 | 12.23658 |
| 661 | 2.92305 | 12.23784 |
| 662 | 2.92741 | 12.23910 |
| 663 | 2.93178 | 12.24034 |
| 664 | 2.93614 | 12.24158 |
| 665 | 2.94050 | 12.24281 |
| 666 | 2.94486 | 12.24404 |
| 667 | 2.94800 | 12.24491 |
| 668 | 2.94923 | 12.24525 |
| 669 | 2.95359 | 12.24646 |
| 670 | 2.95795 | 12.24766 |
| 671 | 2.96232 | 12.24885 |
| 672 | 2.96668 | 12.25004 |
| 673 | 2.97104 | 12.25122 |
| 674 | 2.97540 | 12.25239 |
| 675 | 2.97977 | 12.25356 |
| 676 | 2.98413 | 12.25472 |
| 677 | 2.98849 | 12.25587 |
| 678 | 2.99286 | 12.25701 |
| 679 | 2.99722 | 12.25815 |
| 680 | 3.00158 | 12.25928 |
| 681 | 3.00594 | 12.26040 |
| 682 | 3.01031 | 12.26152 |
| 683 | 3.01467 | 12.26262 |
| 684 | 3.01903 | 12.26373 |
| 685 | 3.02339 | 12.26482 |
| 686 | 3.02776 | 12.26591 |
| 687 | 3.03212 | 12.26699 |
| 688 | 3.03648 | 12.26807 |
| 689 | 3.04085 | 12.26914 |
| 690 | 3.04521 | 12.27020 |
| 691 | 3.04957 | 12.27126 |
| 692 | 3.05393 | 12.27231 |
| 693 | 3.05830 | 12.27335 |
| 694 | 3.06266 | 12.27439 |
| 695 | 3.06360 | 12.27461 |
| 696 | 3.06702 | 12.27542 |
| 697 | 3.07138 | 12.27644 |
| 698 | 3.07575 | 12.27746 |
| 699 | 3.08011 | 12.27847 |
| 700 | 3.08447 | 12.27948 |
| 701 | 3.08884 | 12.28048 |
| 702 | 3.09320 | 12.28147 |
| 703 | 3.09756 | 12.28246 |
| 704 | 3.10192 | 12.28344 |
| 705 | 3.10629 | 12.28441 |
| 706 | 3.11065 | 12.28538 |
| 707 | 3.11501 | 12.28635 |
| 708 | 3.11938 | 12.28731 |
| 709 | 3.12374 | 12.28826 |
| 710 | 3.12810 | 12.28920 |
| 711 | 3.13246 | 12.29014 |
| 712 | 3.13683 | 12.29108 |
| 713 | 3.14119 | 12.29201 |
| 714 | 3.14555 | 12.29293 |
| 715 | 3.14991 | 12.29385 |
| 716 | 3.15428 | 12.29476 |
| 717 | 3.15864 | 12.29566 |
| 718 | 3.16300 | 12.29656 |
| 719 | 3.16737 | 12.29746 |
| 720 | 3.17173 | 12.29835 |
| 721 | 3.17609 | 12.29923 |
| 722 | 3.18045 | 12.30011 |
| 723 | 3.18482 | 12.30099 |
| 724 | 3.18918 | 12.30185 |
| 725 | 3.19354 | 12.30272 |
| 726 | 3.19791 | 12.30357 |
| 727 | 3.20227 | 12.30443 |
| 728 | 3.20663 | 12.30527 |
| 729 | 3.21099 | 12.30612 |
| 730 | 3.21390 | 12.30667 |
| 731 | 3.21536 | 12.30695 |
| 732 | 3.21972 | 12.30778 |
| 733 | 3.22408 | 12.30861 |
| 734 | 3.22844 | 12.30943 |
| 735 | 3.23281 | 12.31025 |
| 736 | 3.23717 | 12.31106 |
| 737 | 3.24153 | 12.31187 |
| 738 | 3.24590 | 12.31267 |
| 739 | 3.25026 | 12.31347 |
| 740 | 3.25462 | 12.31426 |
| 741 | 3.25898 | 12.31504 |
| 742 | 3.26335 | 12.31583 |
| 743 | 3.26771 | 12.31660 |
| 744 | 3.27207 | 12.31738 |
| 745 | 3.27643 | 12.31815 |
| 746 | 3.28080 | 12.31891 |
| 747 | 3.28516 | 12.31967 |
| 748 | 3.28952 | 12.32042 |
| 749 | 3.29389 | 12.32117 |
| 750 | 3.29825 | 12.32192 |
| 751 | 3.30261 | 12.32266 |
| 752 | 3.30640 | 12.32330 |
| 753 | 3.30697 | 12.32339 |
| 754 | 3.31134 | 12.32412 |
| 755 | 3.31570 | 12.32485 |
| 756 | 3.32006 | 12.32557 |
| 757 | 3.32443 | 12.32629 |
| 758 | 3.32879 | 12.32700 |
| 759 | 3.33315 | 12.32771 |
| 760 | 3.33751 | 12.32842 |
| 761 | 3.34188 | 12.32912 |
| 762 | 3.34624 | 12.32982 |
| 763 | 3.35060 | 12.33051 |
| 764 | 3.35496 | 12.33119 |
| 765 | 3.35933 | 12.33188 |
| 766 | 3.36369 | 12.33256 |
| 767 | 3.36805 | 12.33323 |
| 768 | 3.37242 | 12.33390 |
| 769 | 3.37678 | 12.33457 |
| 770 | 3.38114 | 12.33523 |
| 771 | 3.38550 | 12.33589 |
| 772 | 3.38987 | 12.33655 |
| 773 | 3.39423 | 12.33720 |
| 774 | 3.39859 | 12.33785 |
| 775 | 3.40295 | 12.33849 |
| 776 | 3.40732 | 12.33913 |
| 777 | 3.41168 | 12.33976 |
| 778 | 3.41604 | 12.34040 |
| 779 | 3.42041 | 12.34102 |
| 780 | 3.42477 | 12.34165 |
| 781 | 3.42913 | 12.34227 |
| 782 | 3.43349 | 12.34288 |
| 783 | 3.43786 | 12.34349 |
| 784 | 3.44222 | 12.34410 |
| 785 | 3.44658 | 12.34471 |
| 786 | 3.45095 | 12.34531 |
| 787 | 3.45531 | 12.34591 |
| 788 | 3.45660 | 12.34608 |
| 789 | 3.45967 | 12.34650 |
| 790 | 3.46403 | 12.34709 |
| 791 | 3.46840 | 12.34768 |
| 792 | 3.47276 | 12.34826 |
| 793 | 3.47712 | 12.34884 |
| 794 | 3.48148 | 12.34942 |
| 795 | 3.48585 | 12.34999 |
| 796 | 3.49021 | 12.35056 |
| 797 | 3.49457 | 12.35112 |
| 798 | 3.49894 | 12.35168 |
| 799 | 3.50330 | 12.35224 |
| 800 | 3.50766 | 12.35280 |
| 801 | 3.51202 | 12.35335 |
| 802 | 3.51639 | 12.35390 |
| 803 | 3.52075 | 12.35444 |
| 804 | 3.52511 | 12.35498 |
| 805 | 3.52948 | 12.35552 |
| 806 | 3.53384 | 12.35606 |
| 807 | 3.53760 | 12.35652 |
| 808 | 3.53820 | 12.35659 |
| 809 | 3.54256 | 12.35712 |
| 810 | 3.54693 | 12.35764 |
| 811 | 3.55129 | 12.35817 |
| 812 | 3.55565 | 12.35868 |
| 813 | 3.56001 | 12.35920 |
| 814 | 3.56438 | 12.35971 |
| 815 | 3.56874 | 12.36022 |
| 816 | 3.57310 | 12.36073 |
| 817 | 3.57747 | 12.36123 |
| 818 | 3.58183 | 12.36173 |
| 819 | 3.58619 | 12.36223 |
| 820 | 3.59055 | 12.36272 |
| 821 | 3.59492 | 12.36321 |
| 822 | 3.59928 | 12.36370 |
| 823 | 3.60364 | 12.36419 |
| 824 | 3.60800 | 12.36467 |
| 825 | 3.61237 | 12.36515 |
| 826 | 3.61673 | 12.36562 |
| 827 | 3.62109 | 12.36610 |
| 828 | 3.62546 | 12.36657 |
| 829 | 3.62982 | 12.36704 |
| 830 | 3.63418 | 12.36750 |
| 831 | 3.63854 | 12.36796 |
| 832 | 3.64160 | 12.36828 |
| 833 | 3.64291 | 12.36842 |
| 834 | 3.64727 | 12.36888 |
| 835 | 3.65163 | 12.36933 |
| 836 | 3.65600 | 12.36978 |
| 837 | 3.66036 | 12.37023 |
| 838 | 3.66472 | 12.37068 |
| 839 | 3.66908 | 12.37112 |
| 840 | 3.67345 | 12.37156 |
| 841 | 3.67781 | 12.37200 |
| 842 | 3.68217 | 12.37243 |
| 843 | 3.68653 | 12.37286 |
| 844 | 3.69090 | 12.37329 |
| 845 | 3.69526 | 12.37372 |
| 846 | 3.69962 | 12.37414 |
| 847 | 3.70399 | 12.37456 |
| 848 | 3.70835 | 12.37498 |
| 849 | 3.71271 | 12.37540 |
| 850 | 3.71707 | 12.37581 |
| 851 | 3.72144 | 12.37622 |
| 852 | 3.72580 | 12.37663 |
| 853 | 3.73016 | 12.37704 |
| 854 | 3.73452 | 12.37744 |
| 855 | 3.73889 | 12.37784 |
| 856 | 3.74325 | 12.37824 |
| 857 | 3.74761 | 12.37864 |
| 858 | 3.75198 | 12.37903 |
| 859 | 3.75634 | 12.37942 |
| 860 | 3.76070 | 12.37981 |
| 861 | 3.76506 | 12.38020 |
| 862 | 3.76943 | 12.38058 |
| 863 | 3.77379 | 12.38096 |
| 864 | 3.77815 | 12.38134 |
| 865 | 3.78252 | 12.38172 |
| 866 | 3.78688 | 12.38210 |
| 867 | 3.79124 | 12.38247 |
| 868 | 3.79560 | 12.38284 |
| 869 | 3.79997 | 12.38321 |
| 870 | 3.80433 | 12.38357 |
| 871 | 3.80869 | 12.38394 |
| 872 | 3.81305 | 12.38430 |
| 873 | 3.81742 | 12.38466 |
| 874 | 3.82178 | 12.38502 |
| 875 | 3.82614 | 12.38537 |
| 876 | 3.83051 | 12.38572 |
| 877 | 3.83487 | 12.38607 |
| 878 | 3.83923 | 12.38642 |
| 879 | 3.84359 | 12.38677 |
| 880 | 3.84796 | 12.38711 |
| 881 | 3.85232 | 12.38746 |
| 882 | 3.85668 | 12.38780 |
| 883 | 3.86105 | 12.38813 |
| 884 | 3.86541 | 12.38847 |
| 885 | 3.86977 | 12.38880 |
| 886 | 3.87413 | 12.38913 |
| 887 | 3.87850 | 12.38946 |
| 888 | 3.88286 | 12.38979 |
| 889 | 3.88722 | 12.39012 |
| 890 | 3.89158 | 12.39044 |
| 891 | 3.89595 | 12.39076 |
| 892 | 3.90031 | 12.39108 |
| 893 | 3.90467 | 12.39140 |
| 894 | 3.90750 | 12.39161 |
| 895 | 3.90904 | 12.39172 |
| 896 | 3.91340 | 12.39203 |
| 897 | 3.91776 | 12.39234 |
| 898 | 3.92212 | 12.39265 |
| 899 | 3.92649 | 12.39296 |
| 900 | 3.93085 | 12.39327 |
| 901 | 3.93521 | 12.39357 |
| 902 | 3.93957 | 12.39388 |
| 903 | 3.94394 | 12.39418 |
| 904 | 3.94830 | 12.39448 |
| 905 | 3.95266 | 12.39477 |
| 906 | 3.95703 | 12.39507 |
| 907 | 3.96139 | 12.39536 |
| 908 | 3.96575 | 12.39565 |
| 909 | 3.97011 | 12.39594 |
| 910 | 3.97448 | 12.39623 |
| 911 | 3.97884 | 12.39652 |
| 912 | 3.98320 | 12.39680 |
| 913 | 3.98757 | 12.39709 |
| 914 | 3.99193 | 12.39737 |
| 915 | 3.99629 | 12.39765 |
| 916 | 4.00065 | 12.39792 |
| 917 | 4.00502 | 12.39820 |
| 918 | 4.00938 | 12.39847 |
| 919 | 4.01374 | 12.39875 |
| 920 | 4.01810 | 12.39902 |
| 921 | 4.02247 | 12.39929 |
| 922 | 4.02683 | 12.39956 |
| 923 | 4.03119 | 12.39982 |
| 924 | 4.03556 | 12.40009 |
| 925 | 4.03992 | 12.40035 |
| 926 | 4.04428 | 12.40061 |
| 927 | 4.04864 | 12.40087 |
| 928 | 4.05301 | 12.40113 |
| 929 | 4.05737 | 12.40138 |
| 930 | 4.06173 | 12.40164 |
| 931 | 4.06609 | 12.40189 |
| 932 | 4.07046 | 12.40214 |
| 933 | 4.07482 | 12.40240 |
| 934 | 4.07918 | 12.40264 |
| 935 | 4.08355 | 12.40289 |
| 936 | 4.08791 | 12.40314 |
| 937 | 4.09227 | 12.40338 |
| 938 | 4.09663 | 12.40362 |
| 939 | 4.10100 | 12.40387 |
| 940 | 4.10536 | 12.40411 |
| 941 | 4.10972 | 12.40434 |
| 942 | 4.11409 | 12.40458 |
| 943 | 4.11845 | 12.40482 |
| 944 | 4.12281 | 12.40505 |
| 945 | 4.12717 | 12.40528 |
| 946 | 4.13154 | 12.40551 |
| 947 | 4.13590 | 12.40574 |
| 948 | 4.13870 | 12.40589 |
| 949 | 4.14026 | 12.40597 |
| 950 | 4.14462 | 12.40620 |
| 951 | 4.14899 | 12.40642 |
| 952 | 4.15335 | 12.40665 |
| 953 | 4.15771 | 12.40687 |
| 954 | 4.16208 | 12.40709 |
| 955 | 4.16644 | 12.40731 |
| 956 | 4.17080 | 12.40753 |
| 957 | 4.17516 | 12.40775 |
| 958 | 4.17953 | 12.40797 |
| 959 | 4.18389 | 12.40818 |
| 960 | 4.18825 | 12.40839 |
| 961 | 4.19262 | 12.40861 |
| 962 | 4.19698 | 12.40882 |
| 963 | 4.20134 | 12.40903 |
| 964 | 4.20570 | 12.40923 |
| 965 | 4.21007 | 12.40944 |
| 966 | 4.21443 | 12.40965 |
| 967 | 4.21879 | 12.40985 |
| 968 | 4.22315 | 12.41005 |
| 969 | 4.22752 | 12.41026 |
| 970 | 4.23120 | 12.41043 |
| 971 | 4.23188 | 12.41046 |
| 972 | 4.23624 | 12.41066 |
| 973 | 4.24061 | 12.41086 |
| 974 | 4.24497 | 12.41105 |
| 975 | 4.24933 | 12.41125 |
| 976 | 4.25369 | 12.41144 |
| 977 | 4.25806 | 12.41164 |
| 978 | 4.26242 | 12.41183 |
| 979 | 4.26678 | 12.41202 |
| 980 | 4.27114 | 12.41221 |
| 981 | 4.27551 | 12.41240 |
| 982 | 4.27987 | 12.41259 |
| 983 | 4.28423 | 12.41277 |
| 984 | 4.28860 | 12.41296 |
| 985 | 4.29296 | 12.41314 |
| 986 | 4.29732 | 12.41333 |
| 987 | 4.30168 | 12.41351 |
| 988 | 4.30605 | 12.41369 |
| 989 | 4.31041 | 12.41387 |
| 990 | 4.31477 | 12.41405 |
| 991 | 4.31914 | 12.41423 |
| 992 | 4.32350 | 12.41440 |
| 993 | 4.32786 | 12.41458 |
| 994 | 4.33222 | 12.41475 |
| 995 | 4.33659 | 12.41493 |
| 996 | 4.34095 | 12.41510 |
| 997 | 4.34531 | 12.41527 |
| 998 | 4.34967 | 12.41544 |
| 999 | 4.35404 | 12.41561 |
| 1000 | 4.35840 | 12.41578 |

**S3 Fig. Fig 12. Uniaxial compression curves of ice bodies and ballast-ice composites in the laboratory and DEM under different temperature conditions.**

| Laboratory | | | | | | DEM | | | | | |
| --- | --- | --- | --- | --- | --- | --- | --- | --- | --- | --- | --- |
| -10℃ | | -20℃ | | -30℃ | | -10℃ | | -20℃ | | -30℃ | |
| Distance (mm) | Compressive strength (MPa) | Distance (mm) | Compressive strength (MPa) | Distance (mm) | Compressive strength (MPa) | Distance (mm) | Compressive strength (MPa) | Distance (mm) | Compressive strength (MPa) | Distance (mm) | Compressive strength (MPa) |
| 0 | 0 | 0 | 0 | 0 | 0 | 0 | 0 | 0 | 0 | 0 | 0 |
| 0.1 | 0.00676 | 0.1 | 0.07729 | 0.1 | 0.06448 | 0.006 | 0.047 | 0.007 | 0.0736 | 0.006 | 0.072 |
| 0.2 | 0.01743 | 0.2 | 0.13034 | 0.2 | 0.12897 | 0.01 | 0.108 | 0.011 | 0.138 | 0.01 | 0.138 |
| 0.3 | 0.08032 | 0.3 | 0.18424 | 0.3 | 0.19343 | 0.017 | 0.126 | 0.019 | 0.1656 | 0.018 | 0.165 |
| 0.4 | 0.14209 | 0.4 | 0.23891 | 0.4 | 0.25779 | 0.025 | 0.144 | 0.034 | 0.19665 | 0.028 | 0.192 |
| 0.5 | 0.20272 | 0.5 | 0.29424 | 0.5 | 0.32201 | 0.043 | 0.165 | 0.075 | 0.22425 | 0.056 | 0.222 |
| 0.6 | 0.26222 | 0.6 | 0.35016 | 0.6 | 0.38604 | 0.076 | 0.183 | 0.126 | 0.25185 | 0.106 | 0.249 |
| 0.7 | 0.32058 | 0.7 | 0.40656 | 0.7 | 0.44984 | 0.114 | 0.201 | 0.178 | 0.27945 | 0.157 | 0.276 |
| 0.8 | 0.37782 | 0.8 | 0.46336 | 0.8 | 0.51335 | 0.152 | 0.219 | 0.23 | 0.30705 | 0.208 | 0.303 |
| 0.9 | 0.43392 | 0.9 | 0.52044 | 0.9 | 0.57652 | 0.19 | 0.237 | 0.283 | 0.33465 | 0.26 | 0.33 |
| 1 | 0.48889 | 1 | 0.57772 | 1 | 0.6393 | 0.229 | 0.255 | 0.336 | 0.36225 | 0.311 | 0.357 |
| 1.1 | 0.54273 | 1.1 | 0.6351 | 1.1 | 0.70166 | 0.268 | 0.273 | 0.389 | 0.38985 | 0.368 | 0.387 |
| 1.2 | 0.59543 | 1.2 | 0.69249 | 1.2 | 0.76352 | 0.307 | 0.291 | 0.442 | 0.41745 | 0.42 | 0.414 |
| 1.3 | 0.64701 | 1.3 | 0.74978 | 1.3 | 0.82486 | 0.347 | 0.309 | 0.496 | 0.44505 | 0.471 | 0.441 |
| 1.4 | 0.69745 | 1.4 | 0.80688 | 1.4 | 0.88562 | 0.386 | 0.327 | 0.549 | 0.47265 | 0.522 | 0.468 |
| 1.5 | 0.74676 | 1.5 | 0.86369 | 1.5 | 0.94576 | 0.426 | 0.345 | 0.602 | 0.50025 | 0.573 | 0.495 |
| 1.6 | 0.79494 | 1.6 | 0.92012 | 1.6 | 1.00523 | 0.466 | 0.363 | 0.662 | 0.5309 | 0.63 | 0.525 |
| 1.7 | 0.84198 | 1.7 | 0.97607 | 1.7 | 1.06398 | 0.505 | 0.381 | 0.715 | 0.5589 | 0.68 | 0.552 |
| 1.8 | 0.8879 | 1.8 | 1.03145 | 1.8 | 1.12196 | 0.545 | 0.399 | 0.768 | 0.5865 | 0.731 | 0.579 |
| 1.9 | 0.93268 | 1.9 | 1.08617 | 1.9 | 1.17914 | 0.585 | 0.417 | 0.822 | 0.6141 | 0.78 | 0.606 |
| 2 | 0.97633 | 2 | 1.14012 | 2 | 1.23547 | 0.624 | 0.435 | 0.875 | 0.6417 | 0.831 | 0.633 |
| 2.1 | 1.01885 | 2.1 | 1.19323 | 2.1 | 1.2909 | 0.67 | 0.456 | 0.927 | 0.6693 | 0.885 | 0.663 |
| 2.2 | 1.06023 | 2.2 | 1.2454 | 2.2 | 1.3454 | 0.709 | 0.474 | 0.98 | 0.6969 | 0.934 | 0.69 |
| 2.3 | 1.10049 | 2.3 | 1.29654 | 2.3 | 1.39891 | 0.748 | 0.492 | 1.032 | 0.7245 | 0.984 | 0.717 |
| 2.4 | 1.13961 | 2.4 | 1.34656 | 2.4 | 1.45141 | 0.787 | 0.51 | 1.085 | 0.7521 | 1.032 | 0.744 |
| 2.5 | 1.1776 | 2.5 | 1.39539 | 2.5 | 1.50284 | 0.827 | 0.528 | 1.138 | 0.7797 | 1.081 | 0.771 |
| 2.6 | 1.21446 | 2.6 | 1.44294 | 2.6 | 1.55317 | 0.865 | 0.546 | 1.189 | 0.8073 | 1.134 | 0.801 |
| 2.7 | 1.25018 | 2.7 | 1.48913 | 2.7 | 1.60236 | 0.904 | 0.564 | 1.241 | 0.8349 | 1.182 | 0.828 |
| 2.8 | 1.28478 | 2.8 | 1.53388 | 2.8 | 1.65037 | 0.942 | 0.582 | 1.298 | 0.86595 | 1.229 | 0.855 |
| 2.9 | 1.31824 | 2.9 | 1.57711 | 2.9 | 1.69717 | 0.981 | 0.6 | 1.35 | 0.89355 | 1.276 | 0.882 |
| 3 | 1.35057 | 3 | 1.61876 | 3 | 1.74272 | 1.019 | 0.618 | 1.402 | 0.92115 | 1.323 | 0.909 |
| 3.1 | 1.38176 | 3.1 | 1.65875 | 3.1 | 1.78697 | 1.058 | 0.636 | 1.452 | 0.94875 | 1.376 | 0.939 |
| 3.2 | 1.41183 | 3.2 | 1.69702 | 3.2 | 1.82991 | 1.096 | 0.654 | 1.503 | 0.97635 | 1.423 | 0.966 |
| 3.3 | 1.44076 | 3.3 | 1.7335 | 3.3 | 1.87149 | 1.134 | 0.672 | 1.553 | 1.00395 | 1.469 | 0.993 |
| 3.4 | 1.46856 | 3.4 | 1.76812 | 3.4 | 1.91169 | 1.172 | 0.69 | 1.603 | 1.03155 | 1.516 | 1.02 |
| 3.5 | 1.49523 | 3.5 | 1.80084 | 3.5 | 1.95047 | 1.21 | 0.708 | 1.654 | 1.05915 | 1.561 | 1.047 |
| 3.6 | 1.52077 | 3.6 | 1.83159 | 3.6 | 1.9878 | 1.247 | 0.726 | 1.704 | 1.08675 | 1.607 | 1.074 |
| 3.7 | 1.54518 | 3.7 | 1.86032 | 3.7 | 2.02366 | 1.284 | 0.744 | 1.756 | 1.11435 | 1.657 | 1.104 |
| 3.8 | 1.56845 | 3.8 | 1.88699 | 3.8 | 2.05801 | 1.33 | 0.765 | 1.809 | 1.14195 | 1.702 | 1.131 |
| 3.9 | 1.59059 | 3.9 | 1.91155 | 3.9 | 2.09083 | 1.368 | 0.783 | 1.861 | 1.16955 | 1.747 | 1.158 |
| 4 | 1.6116 | 4 | 1.93396 | 4 | 2.1221 | 1.406 | 0.801 | 1.912 | 1.19715 | 1.791 | 1.185 |
| 4.1 | 1.63148 | 4.1 | 1.95418 | 4.1 | 2.15178 | 1.443 | 0.819 | 1.972 | 1.2282 | 1.835 | 1.212 |
| 4.2 | 1.65022 | 4.2 | 1.97217 | 4.2 | 2.17986 | 1.483 | 0.837 | 2.026 | 1.2558 | 1.883 | 1.242 |
| 4.3 | 1.66784 | 4.3 | 1.98791 | 4.3 | 2.20632 | 1.521 | 0.855 | 2.082 | 1.2834 | 1.927 | 1.269 |
| 4.4 | 1.68432 | 4.4 | 2.00137 | 4.4 | 2.23113 | 1.561 | 0.873 | 2.134 | 1.311 | 1.97 | 1.296 |
| 4.5 | 1.69967 | 4.5 | 2.01253 | 4.5 | 2.25428 | 1.601 | 0.891 | 2.188 | 1.3386 | 2.013 | 1.323 |
| 4.6 | 1.71388 | 4.6 | 2.02136 | 4.6 | 2.27575 | 1.64 | 0.909 | 2.242 | 1.3662 | 2.055 | 1.35 |
| 4.7 | 0.724 | 4.7 | 2.02786 | 4.7 | 2.29551 | 1.678 | 0.927 | 2.293 | 1.3938 | 2.101 | 1.38 |
|  |  | 4.8 | 2.03201 | 4.8 | 2.31357 | 1.717 | 0.945 | 2.351 | 1.4214 | 2.143 | 1.407 |
|  |  | 4.9 | 2.03381 | 4.9 | 2.32989 | 1.757 | 0.963 | 2.403 | 1.449 | 2.184 | 1.434 |
|  |  | 5 | 2.03324 | 5 | 2.34447 | 1.8 | 0.981 | 2.46 | 1.4766 | 2.225 | 1.461 |
|  |  | 5.1 | 2.03032 | 5.1 | 2.35731 | 1.841 | 0.999 | 2.521 | 1.5042 | 2.267 | 1.488 |
|  |  | 5.2 | 2.02505 | 5.2 | 2.36838 | 1.88 | 1.017 | 2.581 | 1.5318 | 2.315 | 1.518 |
|  |  | 5.3 | 0.86554 | 5.3 | 2.37768 | 1.924 | 1.035 | 2.646 | 1.56285 | 2.355 | 1.545 |
|  |  |  | -- | 5.4 | 2.3852 | 1.971 | 1.056 | 2.706 | 1.59045 | 2.396 | 1.572 |
|  |  |  | -- | 5.5 | 2.39094 | 2.017 | 1.074 | 2.765 | 1.61805 | 2.438 | 1.599 |
|  |  |  | -- | 5.6 | 2.39489 | 2.058 | 1.092 | 2.83 | 1.64565 | 2.484 | 1.626 |
|  |  |  | -- | 5.7 | 0.72662 | 2.1 | 1.11 | 2.894 | 1.67325 | 2.529 | 1.656 |
|  |  |  | -- |  |  | 2.143 | 1.128 | 2.956 | 1.70085 | 2.569 | 1.683 |
|  |  |  | -- |  |  | 2.186 | 1.146 | 3.046 | 1.72845 | 2.614 | 1.71 |
|  |  |  | -- |  |  | 2.228 | 1.164 | 3.13 | 1.75605 | 2.657 | 1.737 |
|  |  |  | -- |  |  | 2.285 | 1.182 | 3.216 | 1.78365 | 2.697 | 1.764 |
|  |  |  | -- |  |  | 2.328 | 1.2 | 3.291 | 1.81125 | 2.744 | 1.791 |
|  |  |  | -- |  |  | 2.375 | 1.218 | 3.374 | 1.83885 | 2.802 | 1.821 |
|  |  |  | -- |  |  | 2.427 | 1.236 | 3.467 | 1.86645 | 2.917 | 1.848 |
|  |  |  | -- |  |  | 2.472 | 1.254 | 3.568 | 1.89405 | 2.965 | 1.875 |
|  |  |  | -- |  |  | 2.525 | 1.272 | 3.712 | 1.9251 | 3.018 | 1.902 |
|  |  |  | -- |  |  | 2.574 | 1.29 | 3.851 | 1.9527 | 3.075 | 1.929 |
|  |  |  | -- |  |  | 2.627 | 1.308 | 3.996 | 1.9803 | 3.138 | 1.959 |
|  |  |  | -- |  |  | 2.672 | 1.326 | 4.17 | 2.0079 | 3.199 | 1.986 |
|  |  |  | -- |  |  | 2.725 | 1.344 | 4.352 | 2.0355 | 3.258 | 2.013 |
|  |  |  | -- |  |  | 2.795 | 1.365 | 4.582 | 2.0631 | 3.333 | 2.04 |
|  |  |  | -- |  |  | 2.852 | 1.383 | 4.913 | 2.0907 | 3.395 | 2.067 |
|  |  |  | -- |  |  | 2.911 | 1.401 | 5.292 | 2.1183 | 3.492 | 2.097 |
|  |  |  | -- |  |  | 2.979 | 1.419 | 5.478 | 0.7659 | 3.573 | 2.124 |
|  |  |  | -- |  |  | 3.049 | 1.437 |  | -- | 3.656 | 2.151 |
|  |  |  | -- |  |  | 3.115 | 1.455 |  | -- | 3.755 | 2.178 |
|  |  |  | -- |  |  | 3.192 | 1.473 |  | -- | 3.841 | 2.205 |
|  |  |  | -- |  |  | 3.27 | 1.491 |  | -- | 3.95 | 2.235 |
|  |  |  | -- |  |  | 3.35 | 1.509 |  | -- | 4.063 | 2.262 |
|  |  |  | -- |  |  | 3.451 | 1.527 |  | -- | 4.174 | 2.289 |
|  |  |  | -- |  |  | 3.542 | 1.545 |  | -- | 4.289 | 2.316 |
|  |  |  | -- |  |  | 3.667 | 1.563 |  | -- | 4.43 | 2.343 |
|  |  |  | -- |  |  | 3.792 | 1.581 |  | -- | 4.624 | 2.373 |
|  |  |  | -- |  |  | 3.931 | 1.599 |  | -- | 4.835 | 2.4 |
|  |  |  | -- |  |  | 4.073 | 1.617 |  | -- | 5.024 | 2.427 |
|  |  |  | -- |  |  | 4.319 | 1.635 |  | -- | 5.26 | 2.454 |
|  |  |  | -- |  |  | 4.547 | 1.656 |  | -- | 5.575 | 2.481 |
|  |  |  | -- |  |  | 4.815 | 0.274 |  | -- | 5.937 | 2.508 |
|  |  |  | -- |  |  |  |  |  | -- | 6.1855 | 0.938 |

**S4 Fig. Fig 16. Distributions of contact force chains of ballast beds under different freezing conditions.**

| **Ice** | **Freezing depth** | **Average contact force** | **Max contact force** |
| --- | --- | --- | --- |
| — | — | 6.82 N | 26.3 N |
| 5% | 200 mm | 7.95 N | 31.7 N |
| 15% | 300 mm | 9.24 N | 37.2 N |
| 25% | 485 mm | 10.61 N | 42.8 N |

| **Range** | ***w*=0, depth=0** | ***w*=5%, depth=200 mm** | ***w*=15%, depth=300 mm** | ***w*=25%, depth=485 mm** |
| --- | --- | --- | --- | --- |
| **0 – 5 N** | 58.3% | 51.7% | 45.2% | 39.6% |
| **5 – 10 N** | 27.5% | 28.9% | 29.4% | 29.8% |
| **10 – 15 N** | 9.7% | 12.1% | 14.8% | 17.2% |
| **15 – 20 N** | 3.2% | 4.8% | 6.9% | 9.1% |
| **> 20 N** | 1.3% | 2.5% | 3.7% | 4.3% |

**S5 Fig. Fig 17. Characteristics of particle movements, coordination numbers and bonding keys in the ballast beds under different freezing conditions.**

| ***w*** | **Freezing depth** | **Average velocity (mm/s)** | **Max velocity (mm/s)** | **Change rate** | **Initial number** | **Final number** | **Lossing number** |
| --- | --- | --- | --- | --- | --- | --- | --- |
| — | — | 0.31 | 1.98 | 1.91% | — | — | — |
| 5% | 200 mm | 0.23 | 1.96 | 0.98% | 62779 | 62776 | 3 |
| 15% | 300 mm | 0.16 | 1.93 | 0.52% | 194334 | 194327 | 7 |
| 25% | 485 mm | 0.09 | 1.90 | 0.22% | 323186 | 323174 | 12 |

| **Range (mm/s)** | ***w*=0, depth=0** | ***w*=5%, depth=200 mm** | ***w*=15%, depth=300 mm** | ***w*=25%, depth=485 mm** |
| --- | --- | --- | --- | --- |
| 0 ~ 0.4 | 72.6% | 79.3% | 85.1% | 91.4% |
| 0.4 ~ 0.8 | 18.5% | 14.7% | 10.2% | 6.3% |
| 0.8 ~ 1.2 | 6.2% | 4.1% | 3.0% | 1.6% |
| 1.2 ~ 1.6 | 2.0% | 1.5% | 1.2% | 0.5% |
| 1.6 ~ 2.0 | 0.7% | 0.4% | 0.5% | 0.2% |
